# Synaptic NMDA Receptor-Dependent Ca^2+^ Entry Drives Membrane Potential and Ca^2+^ Oscillations in Spinal Ventral Horn Neurons

**DOI:** 10.1371/journal.pone.0063154

**Published:** 2013-04-30

**Authors:** Michael H. Alpert, Simon Alford

**Affiliations:** Department of Biological Sciences, University of Illinois at Chicago, Chicago, Illinois, United States of America; Indiana University School of Medicine, United States of America

## Abstract

During vertebrate locomotion, spinal neurons act as oscillators when initiated by glutamate release from descending systems. Activation of NMDA receptors initiates Ca^2+^-mediated intrinsic membrane potential oscillations in central pattern generator (CPG) neurons. NMDA receptor-dependent intrinsic oscillations require Ca^2+^-dependent K^+^ (K_Ca_2) channels for burst termination. However, the location of Ca^2+^ entry mediating K_Ca_2 channel activation, and type of Ca^2+^ channel – which includes NMDA receptors and voltage-gated Ca^2+^ channels (VGCCs) – remains elusive. NMDA receptor-dependent Ca^2+^ entry necessitates presynaptic release of glutamate, implying a location at active synapses within dendrites, whereas VGCC-dependent Ca^2+^ entry is not similarly constrained. Where Ca^2+^ enters relative to K_Ca_2 channels is crucial to information processing of synaptic inputs necessary to coordinate locomotion. We demonstrate that Ca^2+^ permeating NMDA receptors is the dominant source of Ca^2+^ during NMDA-dependent oscillations in lamprey spinal neurons. This Ca^2+^ entry is synaptically located, NMDA receptor-dependent, and sufficient to activate K_Ca_2 channels at excitatory interneuron synapses onto other CPG neurons. Selective blockade of VGCCs reduces whole-cell Ca^2+^ entry but leaves membrane potential and Ca^2+^ oscillations unaffected. Furthermore, repetitive oscillations are prevented by fast, but not slow, Ca^2+^ chelation. Taken together, these results demonstrate that K_Ca_2 channels are closely located to NMDA receptor-dependent Ca^2+^ entry. The close spatial relationship between NMDA receptors and K_Ca_2 channels provides an intrinsic mechanism whereby synaptic excitation both excites and subsequently inhibits ventral horn neurons of the spinal motor system. This places the components necessary for oscillation generation, and hence locomotion, at glutamatergic synapses.

## Introduction

Throughout the central nervous system oscillatory activity requires the precise temporal activation and inactivation of neurons. This may be achieved through intercellular inhibition, but also through mechanisms intrinsic to individual neurons. The intrinsic neuronal and synaptic properties that are utilized during oscillatory activity shape the behaviors neurons subserve at the network level. Rhythmic behaviors, such as locomotion, require the transformation of an excitatory command from brainstem neurons into an oscillatory output patterns of motoneuron bursting [1]. The precise rhythmic activity emerges by using both synaptic and intrinsic neuronal properties in concert: intrinsic coupling of excitatory and inhibitory conductances within neurons [2] and reciprocally inhibiting synaptic coupling between neurons [3]–[5].

The underlying properties that drive rhythmicity in neurons of the spinal CPG can directly inform how cellular information processing impacts systems level behaviors. The coupling of depolarization-induced Ca^2+^ entry to activation of an outward Ca^2+^-activated K^+^ channel (K_Ca_2 [6] (formerly SK)) [7] contributes to bursting behavior of spinal motor neurons [8]–[11] and profoundly impacts dendritic integration [12], [13] and plasticity [14]–[16] by tempering excitability [7], [12], [17]. Behaviorally, K_Ca_2 channels mediate the inactivation of depolarizing drive to an agonist muscle group. These channels regulate the frequency and stability of the neural rhythm of the CPG by causing a late after-hyperpolarization following action potentials. While this Ca^2+^-mediated regulation is common across vertebrates [2]–[5], [11], [18]–[23], the route of Ca^2+^ entry, its cellular location, and any synapse-specificity remains undetermined within the spinal motor system. Dendritic Ca^2+^-dependent K^+^ channels are activated by both VGCCs [24], [25] and NMDA receptors [17], [26]–[28] in multiple cell types and brain regions. In lamprey spinal neurons, the contributing routes of Ca^2+^ entry include VGCCs and NMDA receptors, but not release from internal stores [29]. VGCC-mediated Ca^2+^ entry has been shown to activate K_Ca_2 currents involved in the action potential late after-hyperpolarization [30], while progressive Ca^2+^ entry through NMDA receptors may terminate motor burst plateau potentials [8] in non-spiking conditions [2] by activation of K_Ca_2 channels [31].

Determining the route, location, synapse and corresponding proximity of Ca^2+^ entry to K_Ca_2 channels leading to the repolarization of the oscillation is important for understanding how information processing occurs in single neurons [24], [32], particularly within the context of motor control. During NMDA-evoked fictive locomotion, intracellular Ca^2+^ oscillations in lamprey spinal neurons occur in phase with membrane potential oscillations and ventral root bursting [2]–[5], [11], [18]–[23], [33], [34]. These Ca^2+^ oscillations can occur subthreshold to action potential activation and are of greatest amplitude in distal dendrites [33] where NMDA receptors may show greater activation during locomotion [30], [35]. Furthermore, spiking in the same neurons, and presumably VGCC activation, selectively increases Ca^2+^ in the soma, but not in the dendrites [33]. Although K_Ca_2 currents mediated by action potential-driven VGCC activation have profound effects on locomotion [8], [11], [20], [30], [36]–[38], they may be activated in a spatiotemporally different context that does not contribute to oscillations. Thus, we hypothesize that NMDA receptor dependent Ca^2+^ entry underlies repolarization during membrane potential oscillations.

NMDA receptor-dependent Ca^2+^ entry coupled to K_Ca_2 activation [12], [17], [26] will provide a substantially different computational outcome than if activated by VGCCs [25] due to differing cellular function, distribution, response to neuromodulation [12], [39], current generation, and kinetics [40]. VGCCs are invariably activated in response to depolarization, and may be distributed in all regions of the neuronal membrane while varying in subtype [41]–[46] and function [13], [47], [48]. In contrast, NMDA receptors can initiate depolarization and are only physiologically active where synaptically driven by released glutamate. Consequently, NMDA receptors can restrict Ca^2+^ entry to the specific synapse or input dendrite, acting locally as coincidence detectors, coupled to presynaptic activity. Lastly, NMDA receptor-dependent synaptic plasticity allows for the modulation of dendritic signaling. We now demonstrate that the synaptic coupling of NMDA receptor-dependent Ca^2+^ entry to K_Ca_2 activation underlies oscillatory properties of ventral horn neurons in the lamprey spinal cord responsible for locomotor pattern generation.

## Methods

### The Lamprey Preparation

Experiments were performed on isolated spinal cords of late stage larval (voltage clamp step experiments) and recently transformed lampreys (*Petromyzon marinus*). The animals were anesthetized with tricaine methanesulfonate (MS-222; 100 mg/l; Sigma, St. Louis, MO), decapitated, and dissected in cold saline solution of the following composition (in mM): 130 NaCl, 2.1 KCl, 2.6 CaCl_2_, 1.8 MgCl_2_ or 1.8 MgSO_4_, 4 glucose, 5 HEPES, adjusted to a pH of 7.60 with NaOH and to a final osmolarity of 270±5 mOsm. The spinal cord was isolated, removed from the protective *meninx primitiva* and placed in a cooled, small-volume chamber with a sylgard (Dow Corning, Midland, MI) floor that is inserted onto the stage of an upright microscope. At minimum, a 15-segment section of spinal cord was pinned to the sylgard. In experiments involving whole-cell patch recording, a 10–20 µm slice of tissue was removed from the surface of the spinal cord over the ventral horn using a vibratome tissue slicer. Patch pipettes were then readily introduced to the cut ventral surface [49].The recording chamber was continually superfused with cold (8–10°C), oxygenated solution. Solutions of pharmacological agents were bath-applied at a perfusion rate of ∼1 ml/min. TTX, AP5, NBQX, UCL 1684, BAPTA and apamin were purchased from Tocris (Bristol, UK), ω-conotoxin MVIIC was purchased from Alomone labs (Jerusalem, Israel), and all other chemicals were purchased from Sigma (St. Louis, MO). UCL1684 was dissolved in DMSO prior to a 1000-fold dilution (at minimum) in recording solution, and all other drugs were diluted in water to the appropriate stock concentration before achieving final concentration in the perfusion solution. For peptide toxins, bovine serum albumin was added to the solution to a final concentration of 0.1% w/v to prevent binding to the perfusion tubing and facilitate drug delivery to the tissue. During experiments involving NMDA receptor activation, glycine was added as co-agonist for the NMDA receptor to compensate for washout, while strychnine was used to prevent glycine receptor activation.

### Electrophysiology

Ventral horn neurons (motoneurons or interneurons) were recorded in whole-cell voltage clamp configuration, blind to cell identity. The patch pipette solution contained (in mM): 102.5 cesium or potassium methane sulfonate, 1 NaCl, 1 MgCl_2_, 5 EGTA or 9.5 BAPTA, 5 HEPES, 0.3 GTP and 0.3 ATP, pH adjusted to 7.2 with CsOH (for voltage clamp experiments) or KOH (for oscillation and evoked EPSC experiments) to a final osmolarity of 250±2 mOsm. In voltage clamp experiments, 1 mM phosphocreatine (Sigma; St. Louis, MO) was added to the patch solution to prevent rundown of VGCCs. Cell types were identified by their location in the tissue and the electrode positioned on individual neurons under visual guidance. Pipettes had open-tip resistances of 5–10 MΩ. Series resistance compensation was performed for voltage step experiments and was monitored continuously by giving a 10 mV voltage step before each episode. Cells were discarded if the series resistance changed by more than 10%. Voltage steps were given at 5 s intervals to minimize current rundown. After whole-cell access was achieved, 10 min was allowed for diffusion of Ca^2+^ buffers from the pipette (unless stated otherwise) or replacement of Ca^2+^ with Ba^2+^ in the extracellular solution to allow currents to reach maximum amplitude before drug application. Due to the large size of ventral horn neurons in recently transformed lampreys, neurons from larval lampreys were used to minimize space clamp error generated from voltage steps. As an exception, transformer neurons were used for all experiments where membrane NMDA receptor-dependent membrane potential oscillations were induced in current clamp prior to switching to voltage clamp to perform step protocols. In adult transformer lampreys, paired recordings were made between presynaptic reticulospinal (RS) axons recorded with sharp microelectrodes and postsynaptic spinal neurons recorded under whole-cell voltage clamp in Mg^2+^-free ringer (to circumvent NMDA receptor block) while clamping between −70 mV and −60 mV. Sharp microelectrode were made conventionally with thin-walled glass with tip resistances of 20–50 MΩ. The pipette solution for recording and stimulating RS axons was 3 M KCl unless otherwise stated. Action potentials were evoked in the presynaptic axons at 15 s intervals to prevent postsynaptic current rundown. Drugs were only added after a stable baseline was achieved (10–15 min).

### Extracellular Stimulation

Selective, extracellular excitatory interneuron (EIN) stimulation was achieved using custom made tungsten stimulating electrodes. Tungsten wire (A-M Systems, Inc., Sequim, WA) was electrochemically etched to make fine tips, inserted into a glass capillary tube and then secured in place by glue. The glass was then pulled using an electrode puller to expose the tips and sylgard was used for added insulation, only exposing the very tip of the wire. EPSCs were evoked using an isolated stimulator (A360; World Precision Instruments, Sarasota, FL) to deliver low intensity (5–10 µA), highly localized stimulation, 1–2 segments rostral and ipsilateral to the whole-cell voltage clamped cell. Strychnine was simultaneously applied to prevent glycinergic inhibition from coincident stimulation of inhibitory interneurons. Selective EIN excitation and reticulospinal axon avoidance was achieved through several factors: (1) reticulospinal axons have a very high membrane capacitance and require substantial focal current injection to induce an action potential. This means that the axons require more charge than the tungsten electrode delivers at low stimulation intensity, (2) extracellular stimulation is insufficient to cause spikes in reticulospinal axons recorded with a microelectrode, and (3) the majority of reticulospinal axons passing over cell somata are removed from the slice preparation, while remaining medial or lateral axons can be visually avoided. Stimuli were given at 15 s intervals to prevent postsynaptic current rundown and drugs were only added after a stable baseline was achieved (10–15 min).

### Selective Retrograde Labeling of Motoneurons from the Muscle Wall

Recently transformed lampreys were anesthetized. The dextran-conjugated Ca^2+^-sensitive dye, 10,000 MW Oregon Green 488 BAPTA-1 dextran (Life Technologies (Invitrogen), Grand Island, NY), was injected into the body musculature (5 mM, 5 µl) to allow uptake into motoneuron axon terminals. The animal was then allowed to recover in a cooled aquarium, allowing the dye time to be retrogradely transported into somata and dendrites. After 48 h, the animal was decapitated under anesthesia, and the spinal cord adjacent to the injection site was removed. Imaging revealed Ca^2+^-sensitive dye labeling selectively in motoneurons of corresponding hemisegments.

### Imaging

Recently transformed lampreys were used for all imaging experiments. Confocal imaging was performed using a modified Bio-Rad (Hercules, CA) MRC 600 confocal microscope. Two laser excitation wavelengths were used (488 nm argon ion and 568 nm krypton–argon) through an acousto-optic tunable filter-coupled fiber optic launch (Prairie Technologies, Madison, WI). Excitation was applied through a custom dichroic mirror with sharp excitation bands matching the two laser wavelengths (Omega Optical, Brattleboro, VT). Two detectors were placed after a second dichroic, with a transmission band from 500–560 nm and long-pass reflection from 580 nm. Emission filters were bandpass (500–560 nm) and long pass (above 580 nm). The photomultiplier outputs were amplified with low-noise current amplifiers (Stanford Research Systems, Sunnyvale, CA) and digitized to 12 bits with a National Instruments (Austin, TX) board and custom software written under Matlab (Mathworks; Natick, MA). The scan-head mirrors were driven though the MRC 600 scan-head amplifiers with the same custom software, available on our website (http://alford.bios.uic.edu/Research/software.html).

Simultaneous fluorescence imaging combined with electrophysiological recordings were performed with a CCD system (ORCA; Hamamatsu, Hamamatsu City, Japan) mounted onto a compound microscope (Olympus BX50WI; Olympus, Tokyo, Japan) equipped with a rapidly switchable Xenon source (Sutter Lambda DG4, Sutter Instrument Company, Novato, CA) and emission filter wheel (Sutter Lambda 10–2, Sutter Instrument Company, Novato, CA). A 490 nm or 560 nm bandpass excitation filter and 510 nm long pass or 645 nm bandpass emission filter was used for 488 nm Ca^2+^-sensitive dyes, OGB1 and Fluo-5F, or a 568 nm inert dye, respectively. All dyes were purchased from Life Technologies (Invitrogen). Acquisition was performed using µManager software [50], binning images at 1×1, 2×2, and 4×4 as needed.

### Data Analysis

Electrophysiological analysis was performed offline using Axograph X software (Sydney, AU). Imaging data analysis was performed using ImageJ (NIH; Bethesda, MD). For analysis of Oregon Green 488 BAPTA-1 and Fluo-5F, fluorescence intensities after background subtraction were normalized to the baseline (prestimulus), giving a baseline value of *ΔF/F  = 0*. Baseline was defined as the fluorescence at the location analyzed prior to electrical stimulation. For oscillation imaging experiments, this was defined as the fluorescence prior to NMDA application. For figures displaying the location of changes in fluorescence, the entire image sequence was divided by the average of the prestimulus images. This generated a relative fluorescence sequence, the peak of which was averaged and then contrast- and brightness-adjusted to display a black background. For all experiments, cells were discarded if bleaching or response amplitude rundown occurred for the entirety of the control condition. Data are given as means ± SEM and n = number of cells. Student’s paired, one-tailed t test was used to calculate the significance between control and drug conditions.

### Ethics Statement

All experiments on animals were performed in accordance with Institutional and national guidelines as laid down by AALAC and under an approved University of Illinois at Chicago Animal Care Committee protocol issued to SA (Permit #: 12–023). All surgery was performed under MS-222 anesthesia, and all efforts were made to minimize suffering.

## Results

### BAPTA Disrupts NMDA-dependent TTX-resistant Membrane Potential Oscillations

**Figure 1:**
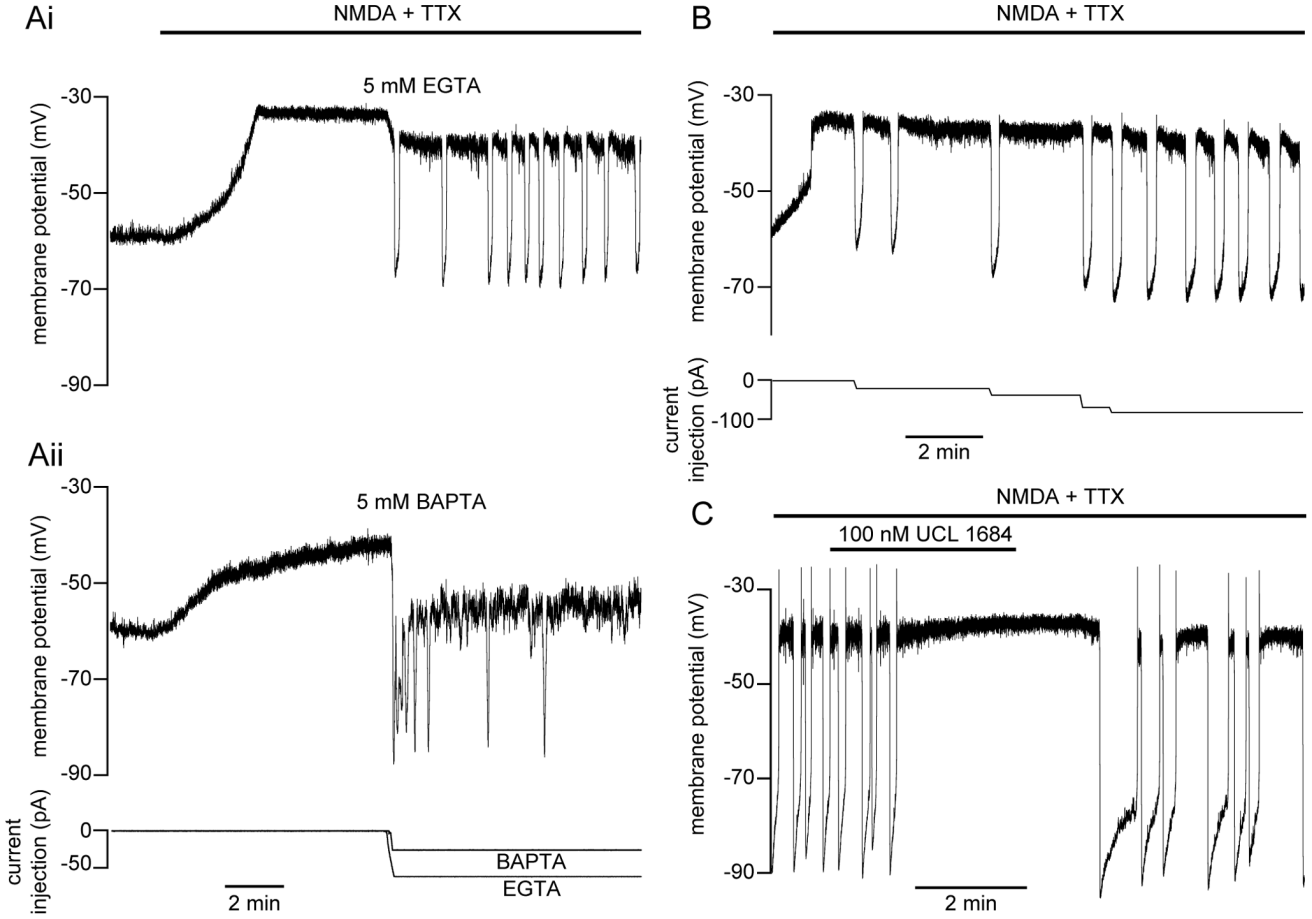
Impairment of NMDA-dependent TTX-resistant oscillations. Following whole-cell access, cells were dialyzed with either EGTA (5 mM) or BAPTA (5 mM) before induction of membrane potential oscillations in NMDA (50 µM), TTX (0.5 µM), glycine (1 µM), and strychnine (5 µM). **A** A ventral horn neuron was dialyzed for 30 minutes prior to NMDA application (black bar). After depolarization, the cell remained at a plateau for several minutes before the minimum amount of negative current necessary for repolarization was injected (bottom trace) and then held constant thereafter. Repetitive oscillations were either induced (EGTA, Ai top trace) or prevented (BAPTA, Aii middle trace). **B** EGTA (5 mM) perfusion immediately following whole-cell access prolongs the oscillation duration, necessitating increasing negative current injection (bottom trace) for repolarization. **C** In a neuron recorded in current clamp with a patch pipette containing EGTA (5 mM), block of K_Ca_2 channels with UCL 1684 (100 nM) prevented NMDA receptor-induced oscillations, which recovered after washout.

NMDA-dependent, TTX-resistant oscillations are Ca^2+^-dependent [2], as Ca^2+^ is necessary for the repolarization of the cell via K_Ca_2 channel activation [8]. However, the route of Ca^2+^ entry is unclear. We hypothesize that oscillations are driven through local, NMDA receptor-dependent synaptic activity where Ca^2+^ entry is coupled to K_Ca_2 channels. To determine if the site of Ca^2+^ entry responsible for repolarization is closely apposed to K_Ca_2 channels, we used whole-cell recordings to dialyze neurons with either BAPTA or EGTA, fast and slow Ca^2+^ buffers, respectively [51], [52]. After 30 min of diffusion following whole-cell access, NMDA (100 µM) and TTX (0.5 µM) were superfused over the spinal cord, depolarizing the cell, which eventually reached a stable plateau (∼ 2 min). Cells were monitored in the depolarized state for several minutes before injecting the minimal amount of negative current necessary to cause a fast hyperpolarization. Cells recorded with EGTA in the patch solution would then autonomously depolarize and subsequently exhibit repetitive membrane potential oscillations (Fig. 1Ai). In contrast, BAPTA (5 mM) within the patch pipette prevents membrane repolarization, abolishing oscillations – cells remain trapped at a depolarized membrane potential (∼−40 mV, Fig. 1Aii; n  = 3). Thus, BAPTA out-competes endogenous buffers to bind entering Ca^2+^ before activating K_Ca_2 channels to repolarize the membrane. In contrast, NMDA (50 µM) invariably induced repetitive oscillations with inclusion of EGTA in the pipette, indicating that EGTA does not bind Ca^2+^ before it can mediate K_Ca_2 channel-dependent repolarization (Fig. 1Ai; 5 mM, n  = 3). However, immediately after whole-cell access, EGTA (0.5–5 mM) diffusion progressively prolongs oscillations (>1 min), requiring increasing levels of current injection to repolarize (n  = 8, Fig. 1B). This indicates that slow Ca^2+^ buffering reduces Ca^2+^ available for K_Ca_2 channel activation, demonstrating that K_Ca_2 channels further away from the site of Ca^2+^ entry can impact the termination of the oscillation. Despite this, the striking effect of BAPTA suggests that K_Ca_2 channels are positioned close to the site of Ca^2+^ entry [51], [52] – BAPTA binds Ca^2+^ rapidly to block K_Ca_2 channel activation whereas EGTA, which binds more slowly, does not. BAPTA’s fast buffering effect is mimicked by blockade of K_Ca_2 channels with the potent inhibitor, UCL 1684 (100 nM). K_Ca_2 channel blockade significantly and reversibly prolonged the oscillation duration (43±18% increase from control; P<0.05, n = 5) and in an additional 2 cells reversibly prevented repolarization (Fig. 1C), confirming that repolarization recorded under whole-cell recording conditions requires activation of K_Ca_2 channels which has previously been shown with apamin in neurons recorded using sharp microelectrodes [8].

### NMDA Modifies the Current-voltage Relationship of Ventral Horn Neurons

**Figure 2:**
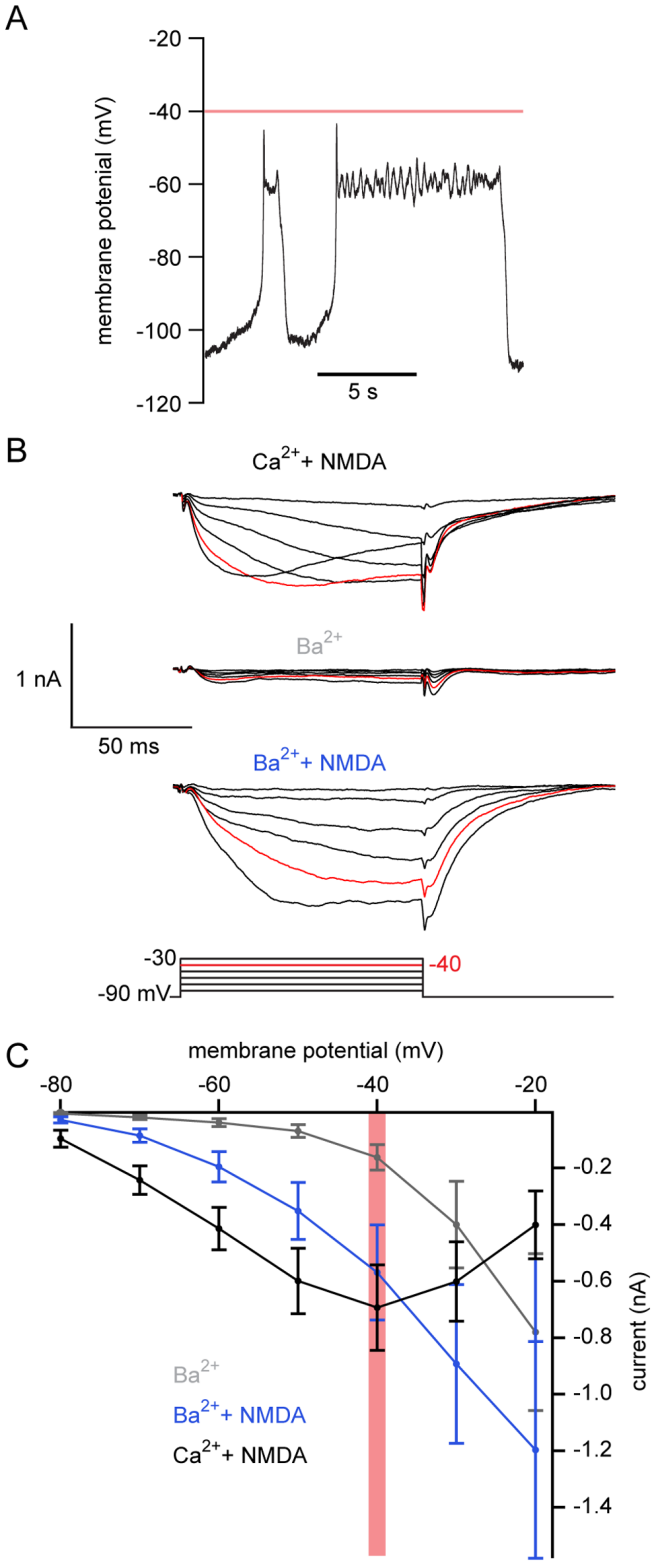
NMDA receptor-induced current entry dominates within the oscillation range. **A.** Oscillations were induced in whole-cell current clamp in a ventral horn neuron (100 µM NMDA, 1 µM TTX) from a recently transformed animal. The average plateau potential in this neuron was −60 mV. **B** The recording mode was switched to voltage clamp in the same cell as A. TEA (5 mM) and 4-AP (1 mM) were added to the solution. A step protocol (10 mV increments from a holding potential of −90 mV) shows the voltage-gated Ca^2+^ current in NMDA (black, 100 µM), following NMDA washout and exchange of Ca^2+^ (2.6 mM) for Ba^2+^ (grey, 2.6 mM). NMDA (100 µM) was then reapplied with Ba^2+^ present (blue). **C** Average I-V plot of voltage-gated currents for Ba^2+^ (grey), Ba^2+^+NMDA (100 µM, blue) and Ca^2+^+NMDA (100 µM, black). Red line and red traces denote −40 mV, the average peak of membrane potential oscillations. Error bars express ± SEM.

Whether the Ca^2+^ responsible for repolarization during oscillations enters the cytosol through NMDA receptors or through VGCCs [34] as the cell is depolarized may be determined by examining the voltage profile of its activation. We determined the membrane potential range over which oscillatory activity occurs and the voltage activation range of channels governing Ca^2+^ influx during oscillations within the same neurons. NMDA (100 µM) and TTX (1 µM) were bath-applied to induce oscillations in whole-cell current clamp (Fig. 2A), determining the peak and trough membrane potentials. Despite differences in oscillation frequency, duration, and trough potential, the peak value reached during the plateau showed little variation between cells (peak = −38.4±1.4 mV, n  = 34), establishing the most depolarized limits of the oscillation membrane potential range.

**Figure 3:**
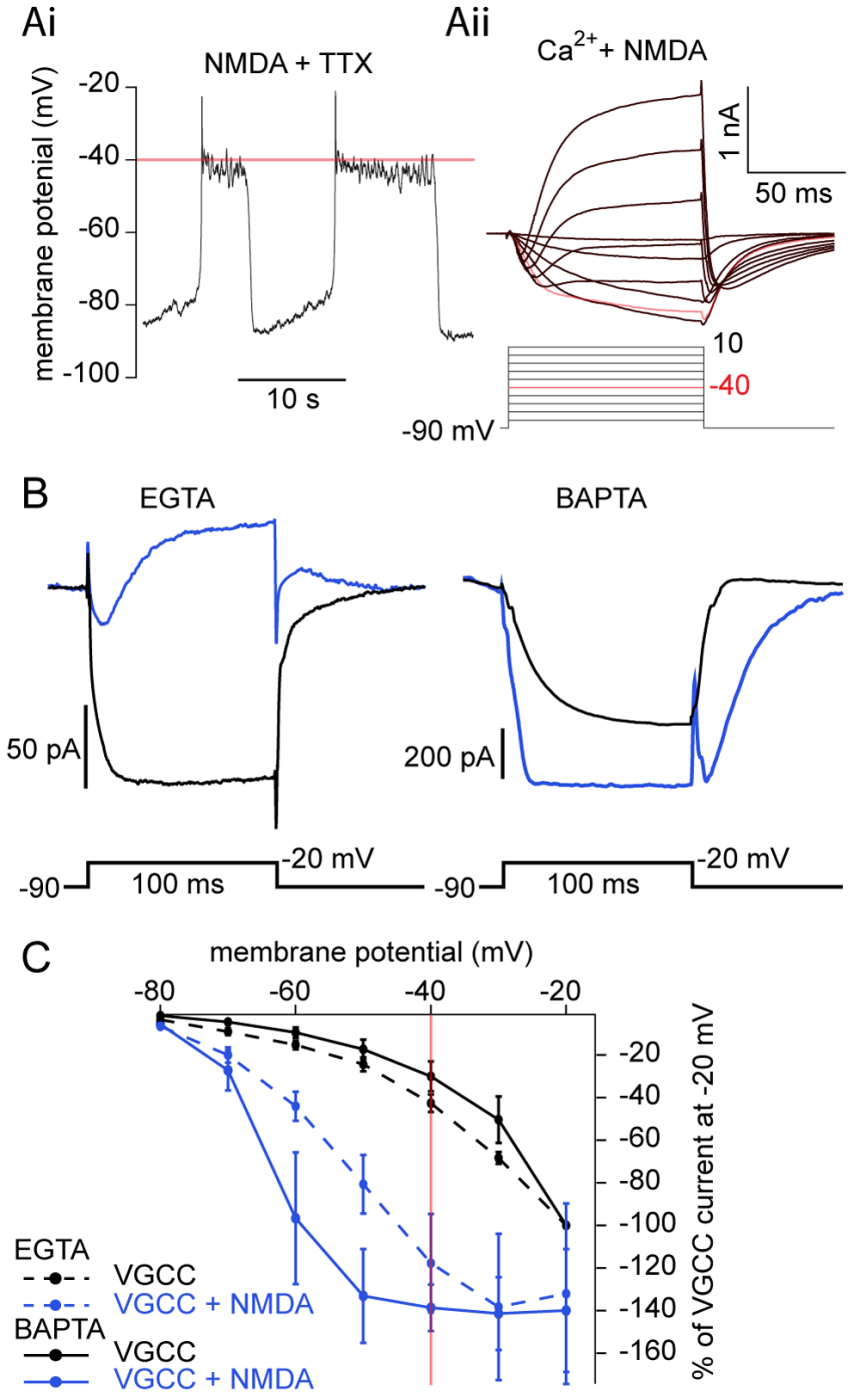
BAPTA prevents an NMDA-induced outward current. Comparison of step-evoked currents between two Ca^2+^ buffers. **A** NMDA (100 µM) and TTX (1 µM) induced oscillations in current clamp (Ai) from a recently transformed lamprey ventral horn neuron dialyzed with EGTA (5 mM). Step-evoked NMDA-receptor dependent current is shown in Aii in the same cell after switching to voltage clamp. K^+^ channels were blocked with bath-application of TEA (5 mM) and 4-AP (1 mM), with Cs^+^ in the patch pipette. With progressive depolarizing steps, NMDA-dependent inward current activation (Aii) becomes biphasic: initial inward currents lead to a subsequent late outward current during a single step. Red line and red traces denote −40 mV, the average peak of membrane potential oscillations. **B** Ventral horn neurons from larval lamprey were whole-cell patched and voltage clamped at −90 mV and then stepped to −20 mV in TTX (1 µM), TEA (5 mM), 4-AP (1 mM), glycine (1 µM), and strychnine (5 µM) before (black) and after the application of NMDA (50 µM, blue). Average current traces in EGTA (5 mM) demonstrate that NMDA leads to the activation of an inward Ca^2+^ current followed by an outward current compared to a purely inward VGCC current. In a different cell, dialysis with BAPTA (9.5 mM) abolishes outward current activation in NMDA, instead potentiating the inward current amplitude relative to VGCCs. **C** Average I-V relationship of cells from B, before (black) and after NMDA (50 µM, blue) application, normalized to peak VGCC current generated at the −20 mV step in EGTA (dotted line) or BAPTA (solid line). NMDA significantly potentiated voltage-dependent currents in BAPTA (−80 to −60 mV) and EGTA (−80 to −40 mV). Red line denotes −40 mV, the average peak of membrane potential oscillations. Error bars express ± SEM.

To examine the relative contribution of Ca^2+^ current from VGCCs or NMDA receptors over the range of membrane potentials observed during oscillations, the same neurons were then switched to voltage clamp at a holding potential of −90 mV while 4-AP (1 mM) and TEA (5 mM) were applied to block voltage-gated K^+^ currents. Depolarizing steps were used to activate voltage-dependent currents in 10 mV increments. Despite blocking K^+^ channels, the true threshold for activation and magnitude of step-evoked inward currents was not measurable with Ca^2+^ in the Ringer’s solution and EGTA as a Ca^2+^ buffer in the recording pipette. A late, secondarily activated, outward current was recorded overlaying the inward current. We hypothesized that this is due to K_Ca_2 channel activation [2], [8], [11], [38], [53]. These currents were prevented by replacing Ca^2+^ (2.6 mM) with Ba^2+^ (1 mM) in the superfusate (Fig. 2B). In Ba^2+^ (Fig. 2B–C; n  = 23) the threshold for VGCC activation was between −40 and −30 mV, which is similar to experiments performed in dissociated lamprey spinal neurons [8], [33], [38], [53]–[55]. In 10 out of 15 cells where oscillations and step protocols were performed in the same cell, VGCC activation thresholds were more depolarized than the average oscillation plateau potential (i.e. −38.4±1.4 mV). The remaining 5 cells activated at −40 mV (n  = 4) or at −50 mV (n  = 1). This indicates that most cells oscillate in a voltage range subthreshold (Fig. 2A), or just approaching (Fig. 3A) the activation threshold of VGCCs.

Addition of NMDA (50–100 µM) to the superfusate hyperpolarized the threshold for current activation to membrane potentials as negative as −80 mV in cells perfused in Ba^2+^ (Fig. 2 B–C; n  = 23) or Ca^2+^ (Fig. 2 C; n  = 14). Due to voltage-dependent unblocking of Mg^2+^ from the NMDA receptor [33], [34], [56], current magnitude increased as the cell was depolarized in NMDA, peaking at −20 mV in Ba^2+^ (Fig. 2C) compared to −40 mV in Ca^2+^ (Fig. 2C). NMDA significantly increased step-induced currents from −80 to −30 mV in Ba^2+^ (P<0.05 at all voltages, n  = 23).

During NMDA-dependent TTX-resistant oscillations (Fig. 3Ai), NMDA receptor activation causes substantial voltage-dependent currents within the oscillation range of ventral horn neurons (Fig. 3Aii). Depolarization to positive potentials leads to progressive outward current activation (Fig. 3Aii). This outward current activation may be due to either Ca^2+^ entry via VGCCs or NMDA receptors. Thus, to further test the hypothesis that NMDA receptor-dependent current activates K_Ca_2 channels within the oscillation range, a comparison was made between cells recorded with EGTA in the patch solution and those recorded with BAPTA. Cells were whole-cell voltage clamped with electrodes containing EGTA (5 mM) at −90 mV and depolarizing steps applied in TTX, 4AP and TEA as above. Again, addition of NMDA leads to a biphasic current in which the initial inward current is followed by activation of an outward current (Fig. 3Aii,B). Depolarizing steps within the NMDA-dependent oscillation range (<−40 mV) failed to activate an outward current without NMDA (i.e. solely VGCCs; Fig. 3B).

With no NMDA present, step-evoked VGCC currents only activated outward currents at very depolarized potentials (more depolarized than −20 mV). Addition of NMDA caused an outward current with steps to −20 mV (Fig. 3B). In contrast, this NMDA-dependent outward current activation was not present in cells dialyzed with BAPTA (9.5 mM; Fig. 3B, n  = 4). NMDA receptor currents are significantly potentiated both in BAPTA (P<0.05; from −80 to −60 mV and −40 to −30 mV; n  = 4) and EGTA (P<0.05, −80 to −40 mV; n  = 6) relative to VGCC-generated current (Fig. 3C). Furthermore, NMDA receptor-dependent currents with BAPTA were more potentiated than with EGTA at more hyperpolarized test potentials (−70 mV to −50 mV). Thus, BAPTA prevents the activation of K_Ca_2 channels in both NMDA-induced membrane potential oscillations in current clamp (Fig. 1Aii) and by step-evoked NMDA receptor activation in voltage clamp (n  = 9; Fig. 3C). This demonstrates that NMDA receptor currents – but not VGCC-dependent currents – are sufficient to activate K_Ca_2 channels in the membrane potential oscillation range.

### NMDA Potentiates Step-evoked Ca^2+^ Signals

**Figure 4:**
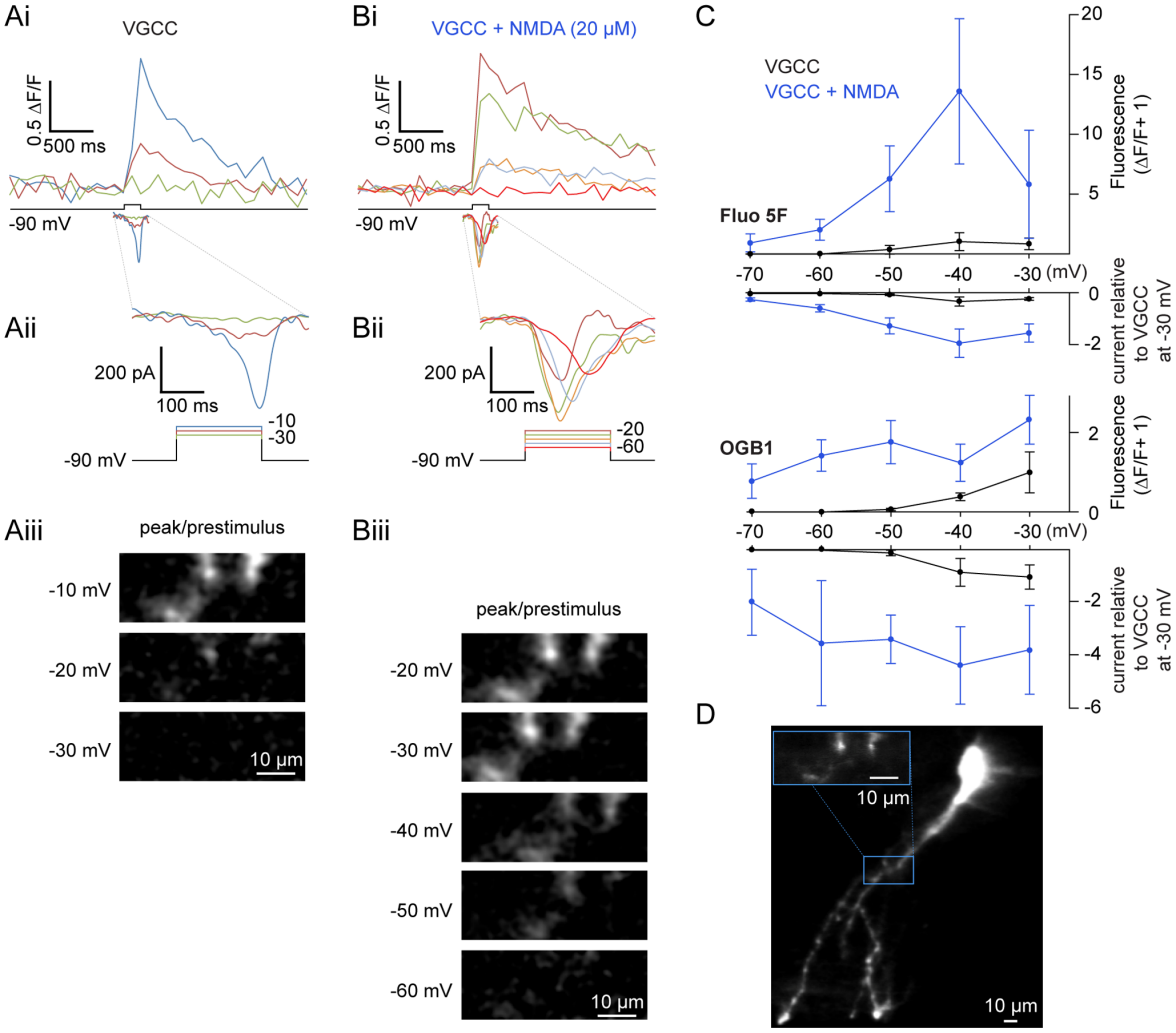
NMDA potentiates step-activated Ca^2+^ currents. Current and fluorescence measurements made from ventral horn neurons filled with Ca^2+^-sensitive dyes administered via the patch pipette. **A–B** In whole-cell voltage clamp, a cell was filled with 200 µM Fluo 5F and 50 µM Alexa Fluor 568 hydrazide. From a holding potential of −90 mV, the cell was depolarized using varying step pulse amplitudes while simultaneously imaging a selected dendritic region using a confocal microscope. VGCC currents (Ai bottom trace, enlarged in Aii) were isolated using TTX (1 µM), TEA (5 mM), and 4-AP (1 mM) before application of NMDA (Bi bottom trace, enlarged in Bii; 20 µM). The corresponding depolarization-induced increase in Ca^2+^ dye fluorescence is shown (top traces, Ai–Bi) on the same time scale as bottom traces (grey box, Ai-Bi). The corresponding changes in fluorescence (displayed as peak/prestimulus fluorescence) at each step are shown below (Aiii, Biii). **C** Pooled step-evoked fluorescence (positive y-axis, expressed as ΔF/F+1) and current (negative y-axis, expressed as a ratio relative to the peak VGCC response at −30 mV) are compared against each voltage step (x-axis) for VGCCs before (black) and after addition of NMDA (blue, 10–100 µM) using either Fluo 5F (top, 100–200 µM) or OGB1 (bottom, 50 µM) as a Ca^2+^-sensitive dye. Error bars express ± SEM. **D** Average z-stack of the cell from A–B filled with Fluo 5F (200 µM) imaged after NMDA application. The inset (blue box) designates the region of analysis; scale bars  = 10 µm.

**Figure 5:**
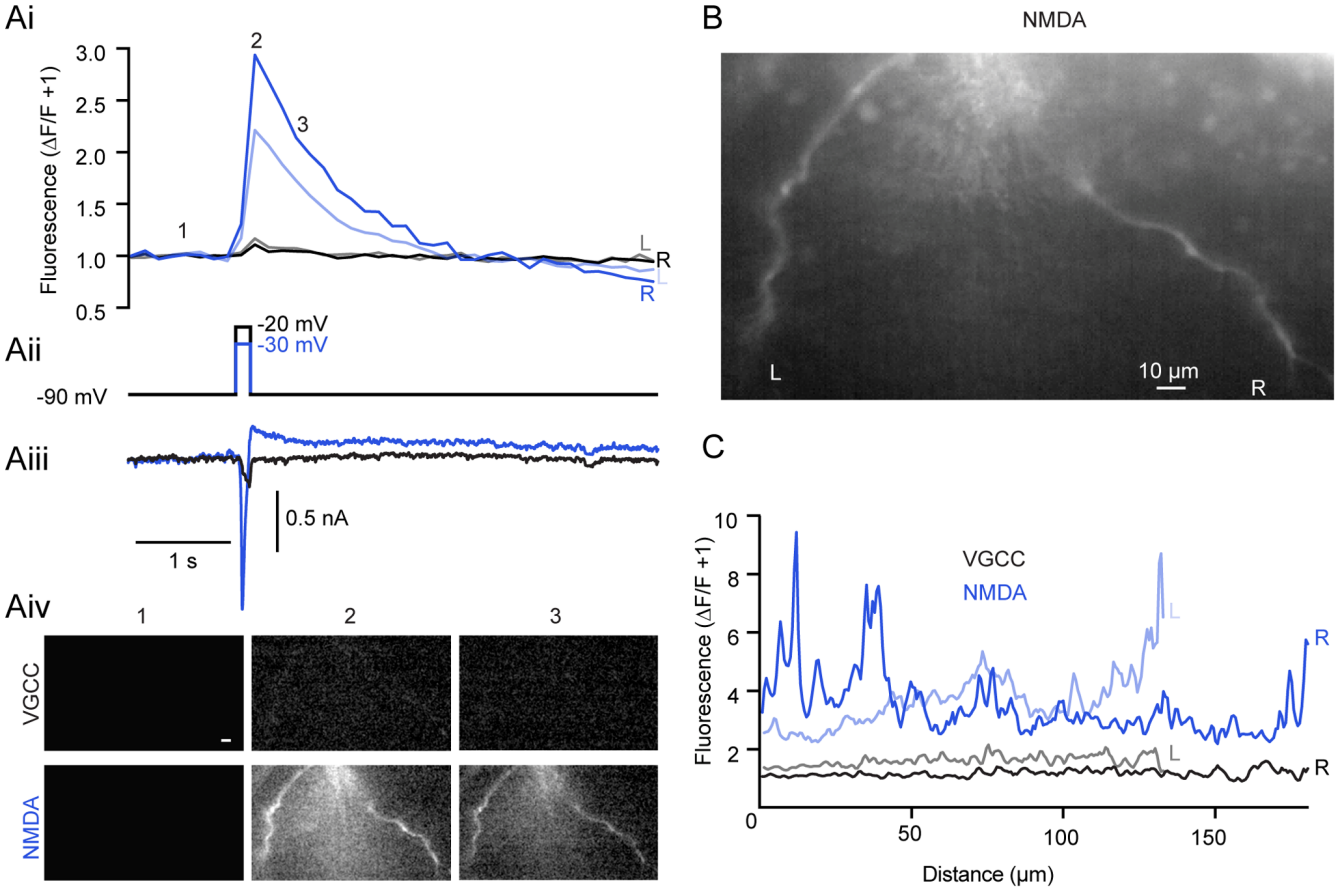
Discrete regions of increased Ca^2+^ fluorescence emerge in NMDA. **A** A cell was whole-cell voltage clamped and dialyzed with Fluo 5F (200 µM) and Alexa Fluor 568 hydrazide (50 µM) and imaged using an epifluorescence CCD system. The cell was then stepped in TTX (1 µM), TEA (5 mM), and 4-AP (1 mM) from −90 mV to −20 mV (Aii, black) to activate VGCC-dependent fluorescence (Ai, black and grey) and current (Aiii, black). After NMDA (50 µM) application, step-evoked (Aii, blue) NMDA receptor-dependent Ca^2+^ fluorescence (Ai, dark and light blue) and current (Aiii, blue) is present at hyperpolarized potentials (Aii, −30 mV, blue). Fluorescent measurements were taken from both left (L; Ai, B, C) and right (R; Ai, B,C) dendritic branches shown in B before (black, grey) and after (blue, light blue) NMDA application. Images showing the location of fluorescent increases taken from the prestimulus (Aiv, 1), peak (Aiv, 2), and decay (Aiv, 3) of the Ca^2+^ signal for VGCCs (Aiv, top row, black) and after NMDA application (Aiv, bottom row, blue) from the same cell expressed as the peak/prestimulus. **B** Raw, unaltered image of the cell in A showing 2 prominent dendritic branches. Scale bar  = 10 µm. **C** Profile of Ca^2+^ signal at response peak along left (L, grey) and right (R, black) dendritic branches (B) from VGCC step to −20 mV (Aii). After NMDA application and step to −30 mV (Aii, blue), left (light blue) and right (dark blue) show both a greater overall fluorescent signal and discrete regions of increased fluorescence along the length of dendrite. 0 µm indicates top of image for both left and right branches.

Whole-cell voltage clamp was combined with Ca^2+^ imaging to determine sites and magnitude of Ca^2+^ entry during voltage steps. Ventral horn neurons were whole-cell clamped with Ca^2+^-sensitive dye (either Oregon Green 488 BAPTA-1 (OGB1) 50 µM; or Fluo-5F, 100–200 µM combined with 50 µM of the inert dye, Alexa Fluor 568 hydrazide) included in the patch solution. Dye was allowed to diffuse into the cell for 30 minutes following whole-cell access and then imaged using a confocal microscope. From a holding potential of −90 mV in TTX (1 µM), 4-AP (1 mM) and TEA (5 mM), cells were stepped to membrane potentials between −70 mV and 0 mV to define the relationship between voltage-dependent currents and Ca^2+^ dye fluorescence (Fig. 4–5). In all dendrites imaged (n  = 15 cells), voltage steps were sufficient to induce fluorescence changes. Thresholds for fluorescence responses evoked by voltage steps are indicated in Table 1. Thus, step-evoked Ca^2+^ signals are activated reliably between −40 and −30 mV via VGCCs, similar to step-evoked currents (Fig. 4A). The ability of OGB1 to detect fluorescence at more hyperpolarized step potentials (e.g., −50 mV, Fig. 4B–C) reflects its higher affinity for Ca^2+^ relative to Fluo 5F (K_d_  = 185 nM and 2.3 µM, respectively) and presumably intracellular Ca^2+^ (Ca^2+^_i_) signals that are too small to be detected by voltage clamp current recording alone in these spatially complex neurons.

**Table 1:** Percent of Cells Showing VGCC-Evoked Ca^2+^ Dye Fluorescence.

Response threshold	−60 mV	−50 mV	−40 mV	−30 mV
Fluo-5F	0% (0/7)	25% (2/8)	63% (7/10)	100% (5/5)
OGB1	0% (0/4)	63% (5/8)	100% (4/4)	100% (3/3)

Similarly to when currents were recorded alone, NMDA (10–100 µM) lowered the threshold for step-evoked Ca^2+^ fluorescence and current. Fluorescent Ca^2+^ signals (Fig. 4B) were evoked at steps to −70 mV with both dyes, subthreshold to VGCC activation (Fig. 4C) in all neurons examined (n  = 15). NMDA also substantially and significantly enhanced step-evoked fluorescence transients previously observed at membrane potentials of −50 mV or more depolarized (Fig. 4C; P<0.05, n  = 8). Using an epifluorescence CCD system, Ca^2+^ signals were also examined in many dendritic regions simultaneously (Fig. 5). Again, NMDA led to a profound increase in both step-evoked fluorescence (Fig. 5Ai) and current magnitude (Fig. 5Aiii). Additionally, NMDA led to discrete regions of enhanced fluorescence along the length of dendrites (Fig. 5B-C). Thus, Ca^2+^-sensitive dyes demonstrate that NMDA receptor activation leads to greater increases in Ca^2+^ entry compared to VGCCs over membrane potential ranges observed during TTX-resistant membrane potential oscillations.

### Effect of Cd^2+^ on Step-evoked VGCC and NMDA Receptor Currents

**Figure 6:**
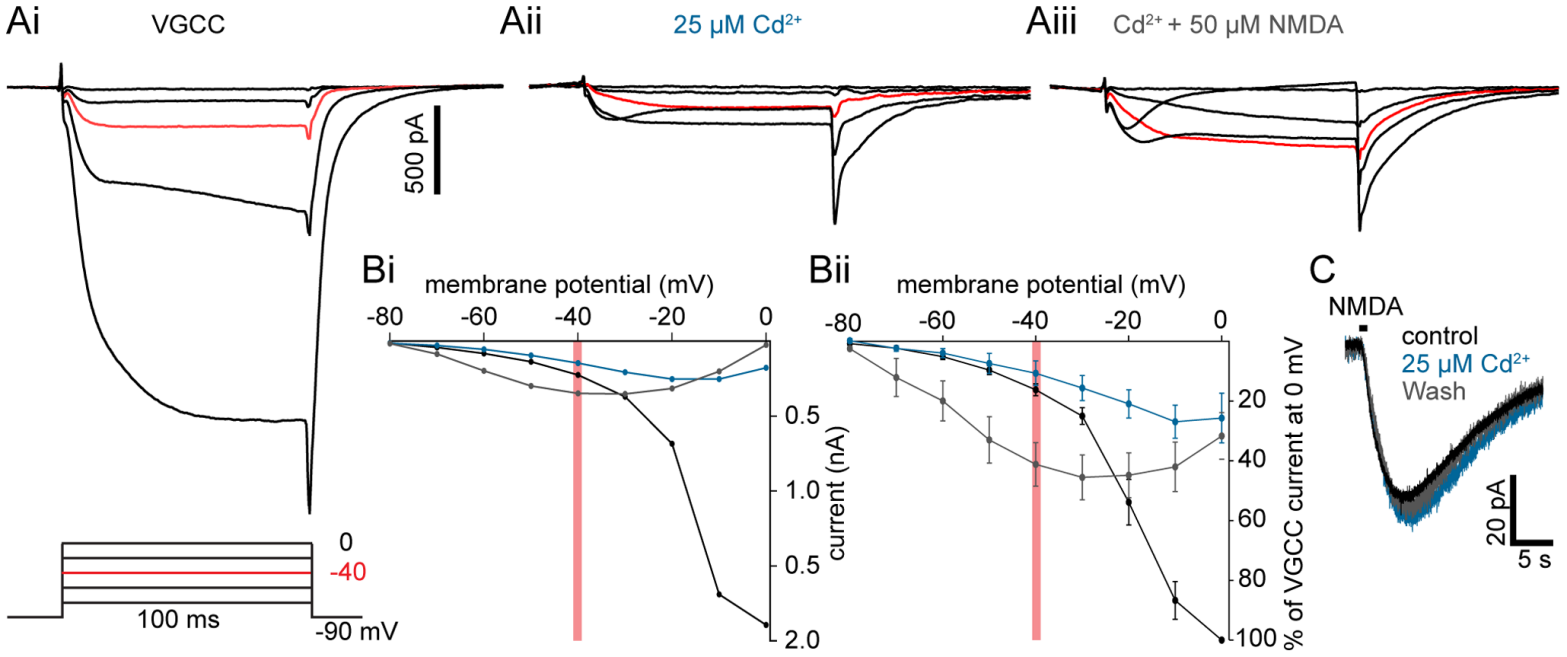
Effect of Cd^2+^ on step-evoked VGCC and NMDA receptor currents. Step protocols performed in cells dialyzed with BAPTA (9.5 mM) demonstrating the effect of Cd^2+^ application to VGCC and NMDA receptor-induced currents. **A** A larval lamprey ventral horn neuron was whole-cell voltage clamped and stepped in TTX (1 µM), TEA (5 mM), and 4-AP (1 mM) from −90 mV to profile VGCC currents (displayed in 20 mV step increments). VGCC currents (Ai, black) are nearly abolished in Cd^2+^ (Aii, teal, 25 µM), but potentiated within the oscillation range (<−40 mV, red line) after NMDA (Aiii, grey, 50 µM) application. Red traces denote −40 mV, the average peak of membrane potential oscillations. **B** I–V plot of cell from A showing voltage-gated Ca^2+^ currents (Bi, black) in the presence of Cd^2+^ (Bi, teal) and NMDA+Cd^2+^ (Bi, grey). An I-V plot of cells pooled from A showing significant potentiation of evoked current in NMDA (Bii, grey) relative to Cd^2+^-depressed currents within the oscillation range, expressed relative to the peak VGCC current at 0 mV. Red line denotes −40 mV, the average peak of membrane potential oscillations. Error bars express ± SEM. **C** A neuron was whole-cell voltage clamped at −70 mV. Ringer’s solution containing NMDA (500 µM) was pressure-ejected (black bar) from a glass pipette (∼5 MΩ) in the approximate vicinity of the dendritic arbor. NMDA receptor EPSCs were recorded before (black), during (teal), and after (grey) the application of Cd^2+^ (25 µM).

To investigate whether VGCC-dependent Ca^2+^ entry is necessary for K_Ca_2-dependent repolarization during membrane potential oscillations recorded in NMDA and TTX, we pharmacologically isolated NMDA receptor-mediated currents. Cd^2+^ was applied as a non-specific high voltage-activated VGCC antagonist during the step protocol. Cd^2+^ completely blocks VGCCs at concentrations of 50–200 µM [35], [57]. However, Cd^2+^ (>50 µM) reduces NMDA receptor currents in cultured rat hippocampal neurons [58], [59]. Thus, to confirm that Cd^2+^ blocks VGCCs at 25 µM *in situ*, neurons were whole-cell voltage clamped (in 1 µM TTX, 1 mM 4-AP, 5 mM TEA) with BAPTA in the patch pipette (9.5 mM) to isolate VGCC currents evoked by stepping from a holding potential of −90 mV as before. Cd^2+^ (25 µM) significantly inhibited VGCC current (Fig. 6Ai–ii,B; inhibited to 25±8% of peak conductance of VGCC at 0 mV, P<0.05, n  = 5). NMDA was then applied to the preparation following Cd^2+^-mediated block. This caused a significant, voltage-dependent potentiation of step-evoked currents (Fig. 6 Aiii,B; −80 to −50 mV, P<0.05, −40 to −30 mV, P<0.01; n  = 5), increasing current magnitude as the cell was depolarized. The resultant current has a similar voltage activation profile to purely NMDA receptor-mediated current. Thus, Cd^2+^ can be used to isolate NMDA receptor-mediated currents with substantial block of VGCCs.

To confirm that this concentration of Cd^2+^ did not cause an inhibition of NMDA receptor- mediated currents [59] in lamprey ventral horn neurons, NMDA (500 µM) was pressure-ejected from a pipette onto neurons recorded in whole-cell voltage clamp at −70 mV to evoke an NMDA receptor-dependent EPSC (Fig. 6C). Bath-applied Cd^2+^ (25 µM) had no effect on either the peak current or area under the curve (P>0.05, n  = 6). At this reduced concentration, Cd^2+^ is thus selective for VGCCs, having little effect on NMDA receptors.

### NMDA-dependent TTX-resistant Oscillations Persist after VGCC Blockade

**Figure 7:**
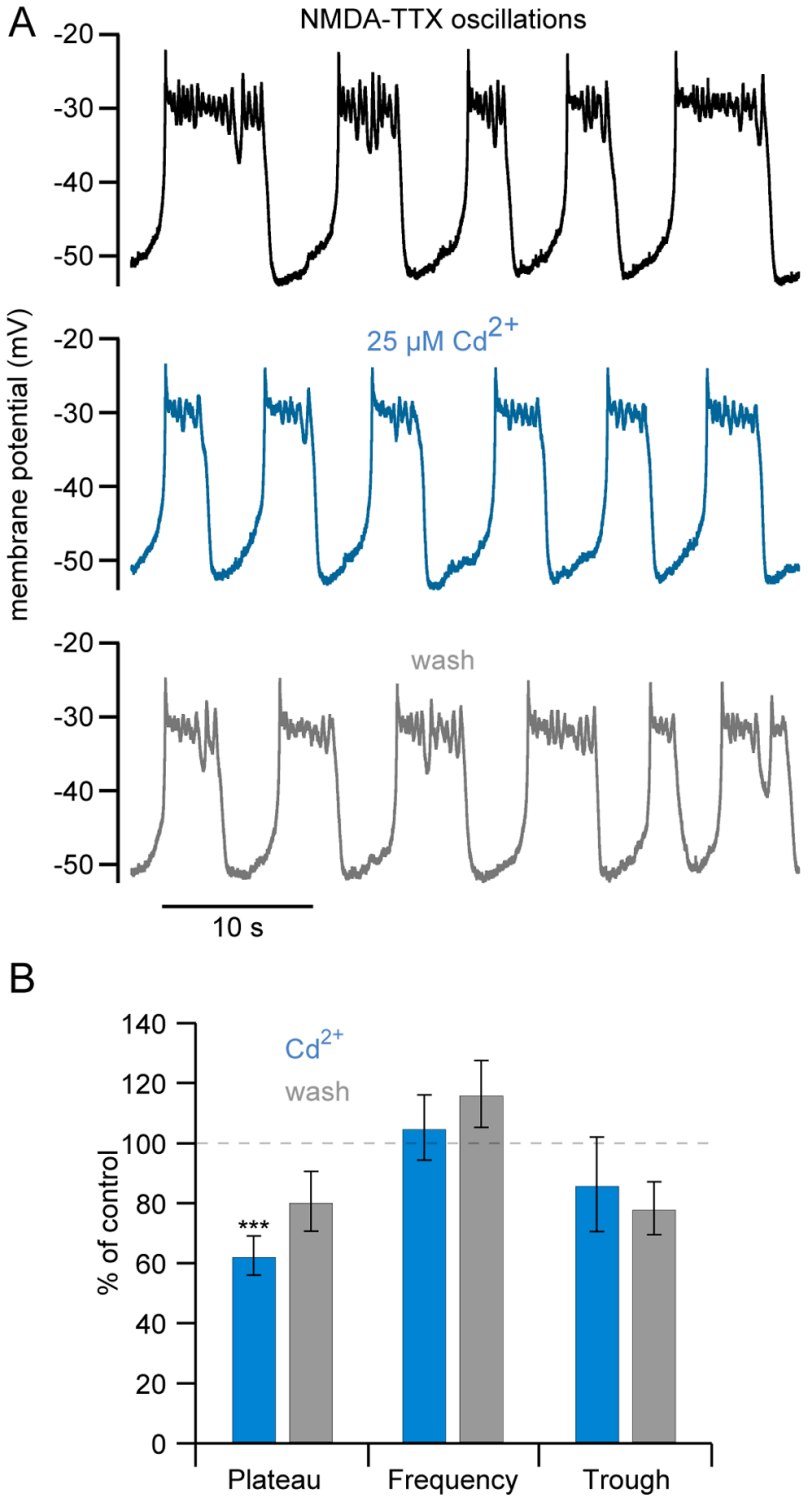
Membrane potential oscillations persist after VGCC blockade. **A** Membrane potential oscillations were induced in a ventral horn neuron in whole-cell current clamp by application of NMDA (50 µM) and TTX (1 µM), buffered with OGB1 (25 µM). Repetitive oscillations (control, black) persisted in the presence of Cd^2+^ (teal; 25 µM) with a small reduction in plateau duration, partially recovering upon washout (grey). **B** Summary of Cd^2+^ effects on oscillation plateau duration, frequency, and trough period duration.

Cd^2+^ was then applied to neurons in which TTX-resistant NMDA receptor-activated membrane potential oscillations were recorded [2] to determine whether VGCCs contribute to the oscillation. Ventral horn neurons were whole-cell current clamped during NMDA (50–100 µM) and TTX (1 µM) application. After dialysis with low concentrations of buffer (25 µM OGB1) and achieving stable induction of membrane potential oscillations (∼5–10 min; Fig. 7A), Cd^2+^ (25 µM) was applied to the superfusate. Cd^2+^ caused a significant and reversible reduction in the duration of the depolarized phase (duration reduced to 62±6.4% of control, *P*<0.001, n  = 12; Fig. 7A,B). In contrast, neither the duration of the hyperpolarized trough period (P>0.05, n  = 8) nor the oscillation frequency showed significant changes (P>0.05, n  = 10) – both measures were variable. Thus, 25 µM Cd^2+^ nearly abolishes step-induced VGCC activation and reduces the duration of depolarization during NMDA receptor-activated membrane potential oscillations. VGCCs may contribute to the depolarizing phase of the oscillation, but do not activate K_Ca_2 channels to influence repolarization.

### Ca^2+^ oscillations Persist after Selective VGCC Block

**Figure 8:**
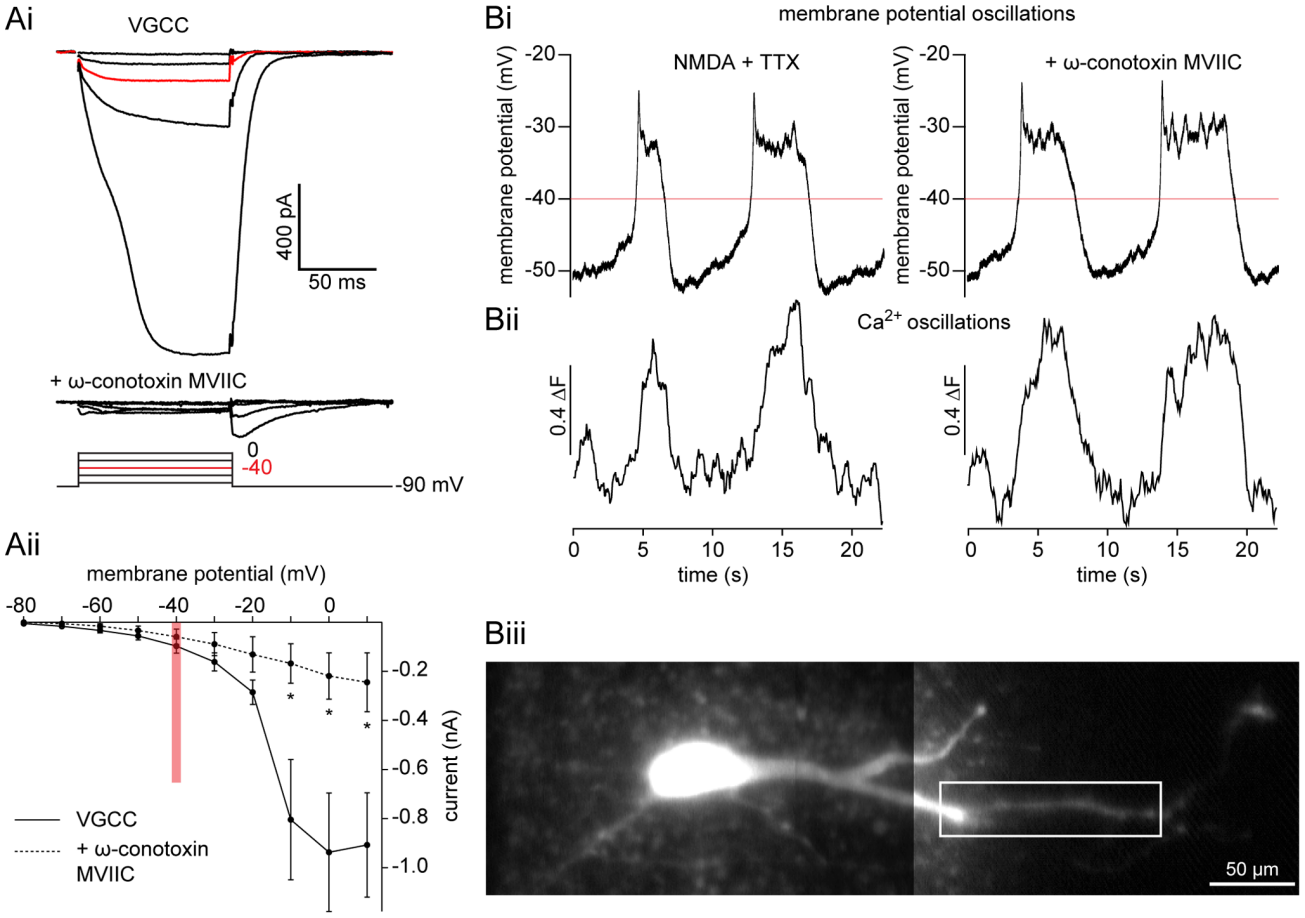
Ca^2+^ oscillations persist after selective VGCC blockade. **A** A ventral horn neuron was whole-cell voltage clamped at −90 mV and stepped (10 mV increments) in TTX (1 µM), TEA (5 mM), and 4-AP (1 mM). Step-induced VGCC current (Ai, top) is blocked by ω-CgTxMVIIC (Ai, bottom; 5 µM). A pooled I-V plot shows the significant reduction of whole-cell VGCC current (Aii, solid line) after application of ω-CgTxMVIIC (Aii, dashed line, 5 µM). Red line and traces denote −40 mV, the average peak of membrane potential oscillations. Error bars express ± SEM. **B** A ventral horn neuron was whole-cell voltage clamped and filled with OGB1 (Biii, 25 µM) at −70 mV. In current clamp mode, application of NMDA (50 µM) and TTX (1 µM) induced simultaneous, repetitive oscillations in membrane potential (Bi) and Ca^2+^ dye fluorescence (Bii, black), which persisted in ω-CgTxMVIIC (Bi-ii; 5 µM). White box denotes region of interest from which measurements in Bii were taken. Red line denotes −40 mV, the average peak of membrane potential oscillations. Scale bar  = 50 µm.

Results obtained using Cd^2+^ indicate that VGCCs do not contribute to repolarization during membrane potential oscillations. However, while Cd^2+^ blocks all VGCC subtypes, it lacks complete selectivity and interferes with Ca^2+^ imaging by binding Ca^2+^-sensitive dyes. Thus, to assess if there is also a reduction in Ca^2+^ entering through VGCCs during membrane potential oscillations, we examined the effect of the specific N- and P/Q-subtype VGCC blocker, ω-conotoxin MVIIC (ω-CgTxMVIIC), on NMDA-dependent TTX-resistant membrane potential and Ca^2+^ oscillations recorded with fluorescent dyes. ω-CgTxMVIIC (5 and 2 µM) inhibits both VGCCs in lamprey cultured ventral horn neurons [60] and the slow afterhyperpolarization in lamprey reticulospinal axons [61]. To confirm that ω-CgTxMVIIC (5 µM) blocks VGCC currents in our *in vitro* spinal cord preparation, we performed membrane potential step protocols as before (in 9.5 mM BAPTA, 1 µM TTX, 1 mM 4-AP, 5 mM TEA). VGCC currents were substantially inhibited to 28±13% of control (Fig. 8A; P<0.05, n  = 3).

To determine whether ω-CgTxMVIIC affects membrane potential and Ca^2+^ oscillations, ventral horn neurons were recorded in current clamp during whole-cell recording with OGB1 (25 µM) in the patch solution. After dye diffusion (∼30 min after whole-cell access), oscillations were induced by bath application of NMDA (50–100 µM) and TTX (1 µM). Image sequences of Ca^2+^ fluorescence in dendrites were acquired while making simultaneous electrophysiological recordings before and during application of ω-CgTxMVIIC (5 µM, Fig. 8Bi). Individual dendritic branches were measured as a single region of interest, comparing mean fluorescence values between each peak and immediately preceding trough. ω-CgTxMVIIC had no effect on the amplitude of fluorescence oscillations (Fig. 8Bii; P>0.05, n  = 5). Despite a change in the membrane potential oscillation waveform in Cd^2+^, there was no significant reduction in the duration due to CgTxMVIIC (P>0.05, n  = 5). Thus, despite a profound block of VGCC current, ω-CgTxMVIIC does not impair membrane potential or Ca^2+^ oscillations.

### Evoked NMDA Receptor EPSCs Lead to Localized Increases in Dendritic Ca^2+^

Intrinsic rhythmicity of individual cells requires K_Ca_2 channel activation. Ca^2+^ entry through either NMDA receptors or VGCCs [33], [34] might activate K_Ca_2 channels. During locomotion, NMDA receptors may be physiologically activated by glutamate release from either descending reticulospinal (RS) neurons [49], [62], or through oscillatory drive provided by local EINs [3], [63].

**Figure 9:**
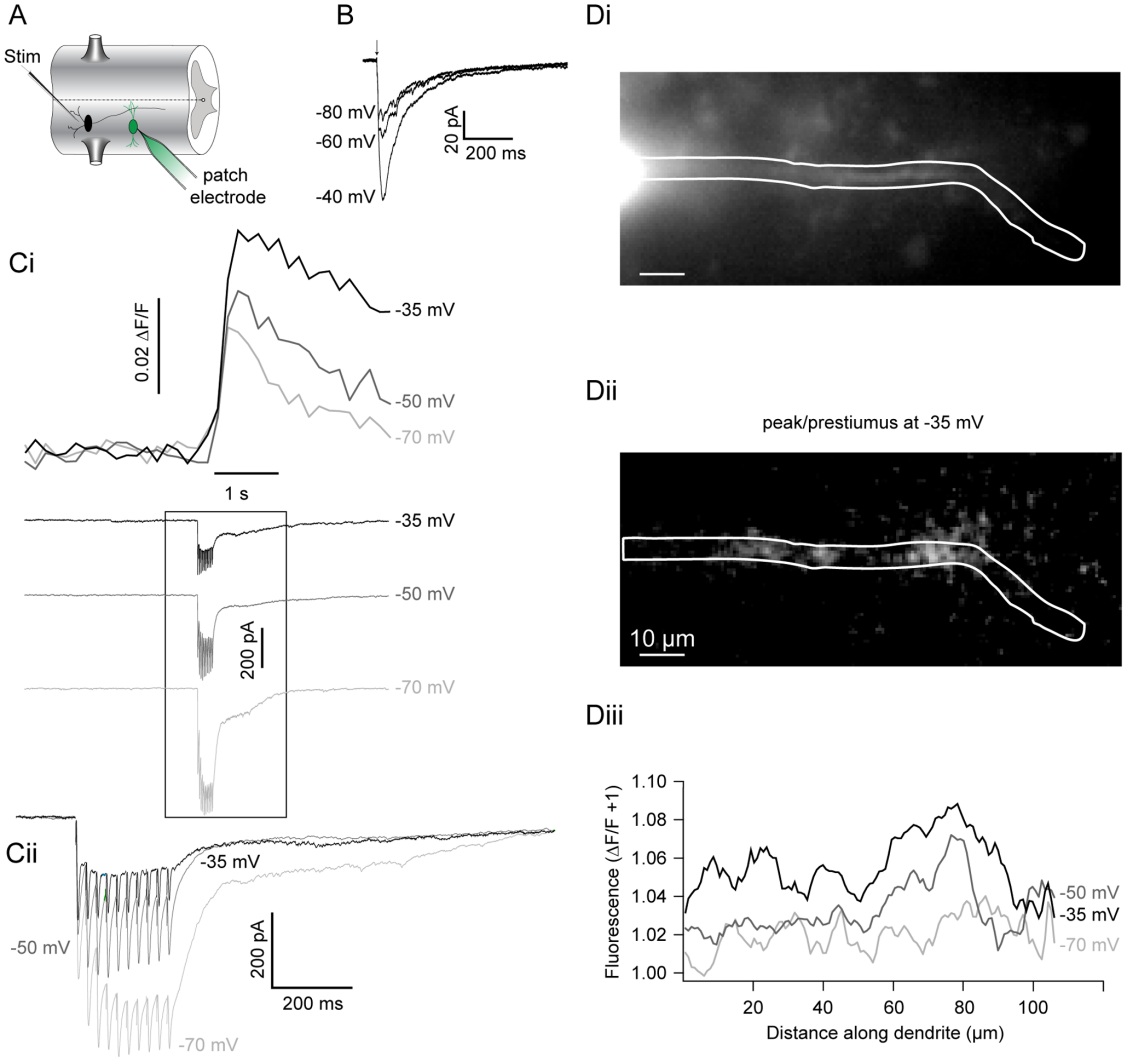
Synaptically evoked Ca^2+^ signals mimic the voltage-dependency of the NMDA receptor current. **A** Recording schematic: STIM = stimulating electrode, EIN = black, Ca^2+^ dye = green. **B** A cell was whole-cell voltage clamped in NBQX (5 µM), glycine (1 µM), strychnine (5 µM) to isolate evoked, NMDA receptor EPSCs, while the holding potential was varied between −80 mV and −40 mV. **C** A ventral horn neuron was whole-cell voltage clamped at −70 mV and filled with OGB1 (50 µM). An extracellular electrode was used to repetitively stimulate (25 Hz for 10 stimuli) excitatory interneurons (EINs) to evoke glutamatergic EPSCs at increasing holding potentials (denoted on right of trace). A transient increase in fluorescence (top) in the dendrite is coincident with the EPSC (bottom). Cii shows an enlargement and EPSC overlay of box in Ci. **D** Image of the ventral horn neuron in C (Di) demonstrating the localization (Dii; white) of evoked fluorescence at −35 mV. Evoked fluorescence changes relative to resting prestimulus fluorescence plotted against dendritic distance at each holding potential (Diii). Scale bar  = 10 µm.

Ventral horn neurons were patched as before with OGB1 (50 µM) included in the patch solution. After dye diffusion, EIN-mediated synaptic responses were selectively recorded by placing a 2–5 µm stimulating electrode, over the ventro-lateral spinal cord (1–2 segments rostral to the patched cell) and using very low intensity stimulation (5–10 µA), which is insufficient to evoke RS axon stimulation (data not shown, see methods). In the presence of strychnine (5 µM), presumed EINs were repetitively stimulated (20–40 Hz; 5–10 stimuli, respectively) to evoke EPSCs recorded somatically in the patched cell while its dendrites were simultaneously imaged. Evoked monosynaptic EPSCs were recorded with an invariant delay from stimulation artifact to peak (Fig. 9A; 10.8±1.6 ms between cells, n  = 6). Stimulation was performed while holding the cell between −70 mV and −35 mV, spanning the region of negative slope conductance of the NMDA receptor-mediated response (Fig. 9B). EPSCs were coincident with an increase in dye fluorescence that far outlasted the EPSC duration (Fig. 9Ci; n  = 8). Low intensity stimulations reliably evoked fluorescent signals that were only found in dendrites, often in discrete locations within the arbor, and with considerable distance from the soma (>100 µm). In cells where the holding potential was varied, a voltage-dependent increase in evoked Ca^2+^ signals was found as the holding potential was increased from −80 mV to −30 mV (Fig. 9Ci). Evoked fluorescent changes at −35 mV were significantly greater than those at −80 mV (13.0±4.5% increase, P<0.05, n  = 6). This confirms that EINs evoke localized Ca^2+^ signals in dendrites within values of membrane potential recorded during NMDA receptor-mediated oscillations.

**Figure 10:**
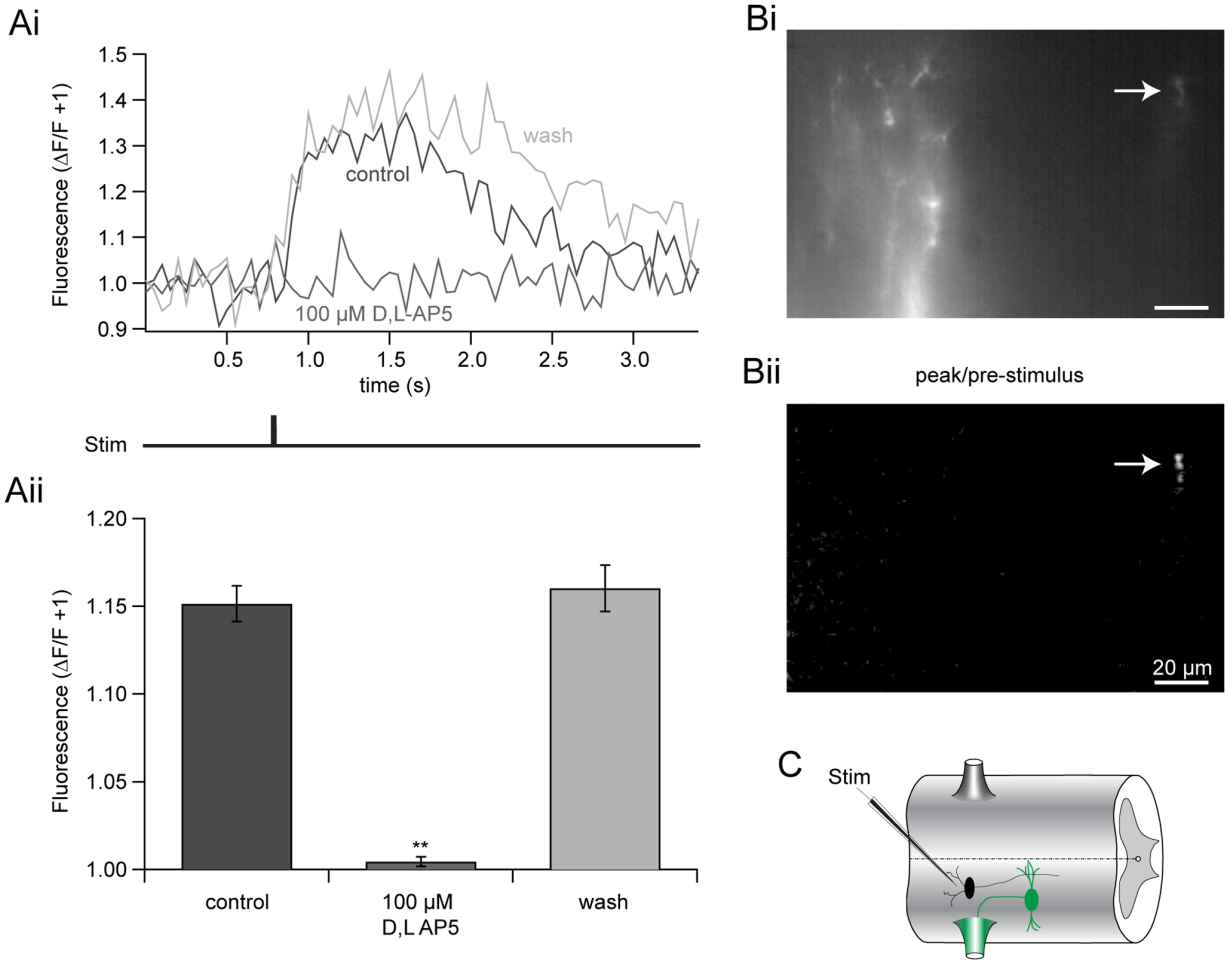
EIN-evoked Ca^2+^ signals are NMDA receptor-dependent. **A** EIN stimulation evoked fluorescent Ca^2+^ signals in a motor neuron dendrite retrogradely filled with the Ca^2+^-sensitive dye, OGB1-dextran (5 mM), in the presence of NBQX (5 µM), glycine (1 µM), and strychnine (5 µM) in Mg^2+^-free ringer. Ca^2+^ signals were recorded before (black), during (dark grey) and after (light grey) the application of D/L-AP5 (100 µM). Pooled responses (Aii) demonstrate that D/L-AP5 significantly and reversibly abolishes NMDA receptor-induced changes in Ca^2+^ fluorescence. Error bars express ± SEM. **B** Image of the dendrite recorded in Ai. Localized EIN-evoked fluorescent change is shown in Bii (arrow, white). Scale bar  = 20 µm. **C** Recording schematic: STIM = stimulating electrode, EIN = black, Ca^2+^-dye filled motor neuron = green.

We confirmed that EIN-evoked, localized Ca^2+^ signals are NMDA receptor-dependent. Motoneurons were retrogradely labeled with the Ca^2+^-sensitive dye, OGB1 dextran applied to ventral roots (see methods). NMDA receptor-mediated responses were pharmacologically isolated with the AMPA receptor antagonist, NBQX (5 µM), and strychnine (5 µM). Single shocks were applied with tungsten microelectrodes (as before), while the extensively labeled dendritic tree of a single motoneuron was simultaneously imaged using a CCD camera. Highly localized, NMDA receptor-dependent Ca^2+^ transients were recorded. Application of the NMDA receptor antagonist, DL-AP5 (100 µM), reversibly abolished these evoked increases in Ca^2+^ fluorescence in all cells imaged (Fig. 10; P<0.05, n  = 5). No reduction in Ca^2+^ transient amplitudes was recorded following NBQX application. Thus, EIN-evoked, localized Ca^2+^ signals are NMDA receptor-dependent.

**Figure 11:**
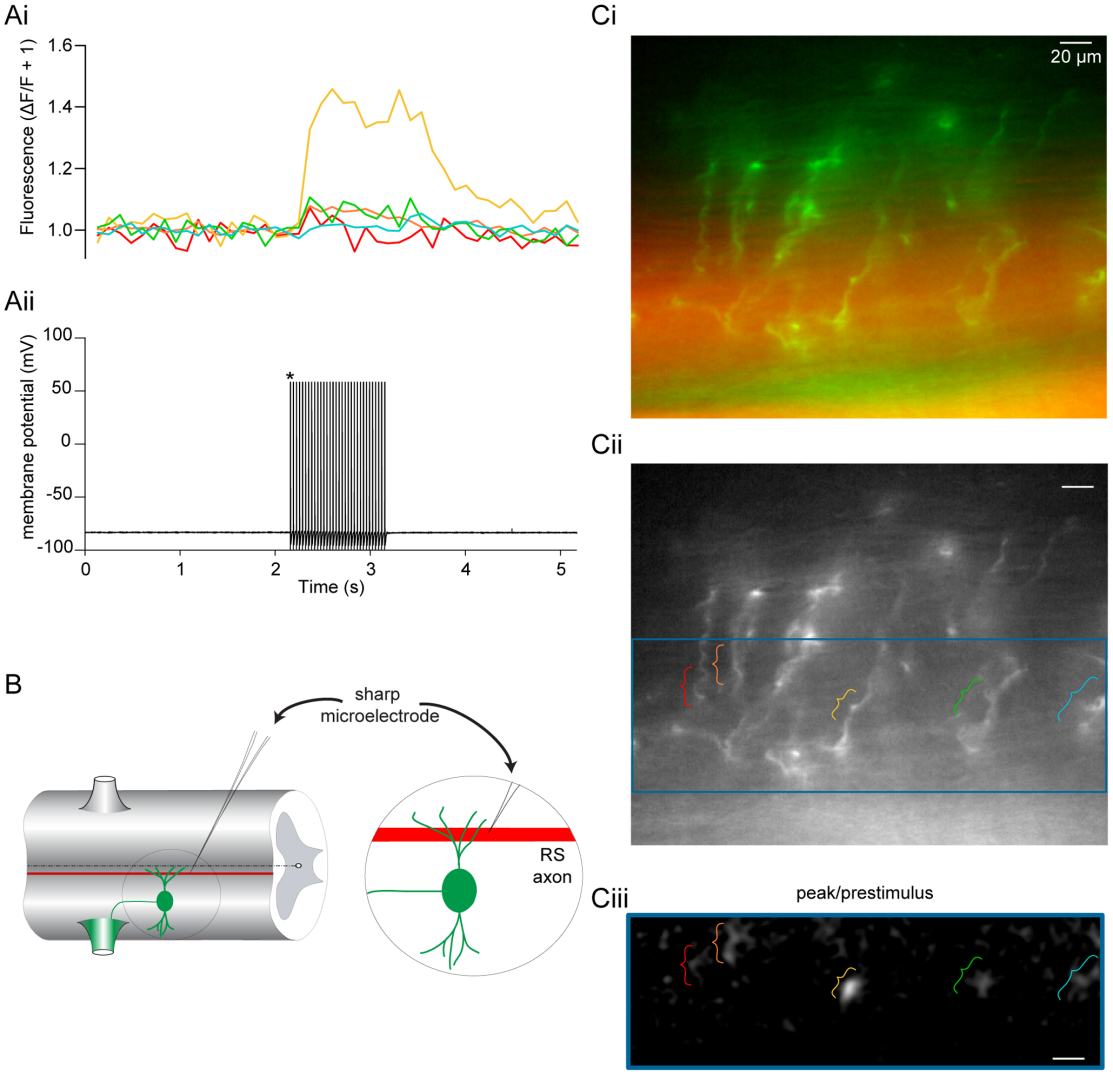
RS axons evoke Ca^2+^ transients in motoneuron dendrites. **A–C** Motoneurons were retrogradely filled via ventral roots with OGB1 dextran (B, green; 5 mM) applied to the trunk musculature. An RS axon passing through the dendritic field was filled with Alexa Fluor 568 hydrazide (B, Ci; red) via pressure ejection and repetitively stimulated (Aii, 32 Hz for 60 s) while simultaneously imaging the postsynaptic dendritic arbor of the filled motor neuron (Ci, green) with a CCD camera in Mg^2+^-free ringer. RS axon action potential firing (Aii) is coincident with dendritic Ca^2+^ transients (Ai). Each color represents the average response from different loci (Cii). A merged image of the dendritic field (Ci; green) is shown with the stimulated axon in addition to another filled axon below (Ci; red). The dendritic region where the axon makes *en passant* synaptic connections is outlined in blue (Cii). The corresponding blue region from Cii shows the location and relative signal intensities measured (Ciii; white), displayed as the peak/prestimulus fluorescence. * Denotes signal truncation for display purposes. Scale bar  = 20 µm.

To determine whether RS axon-mediated excitation also leads to Ca^2+^ entry in ventral horn neuron dendrites, we retrogradely labeled motoneurons with OGB1 dextran to completely fill dendritic arbors (Fig. 11C). Mg^2+^ was removed from the ringer to facilitate NMDA receptor activation and potentiate Ca^2+^ signals for easier detection. A sharp microelectrode filled with the inert dye, Alexa Fluor 568 hydrazide, was used to penetrate, label (Fig. 11C), repetitively stimulate and record from RS axons (Fig. 11Aii) passing through the visualized dendritic arbors (Fig. 11Ci) imaged simultaneously with a CCD camera. Indeed, repetitive RS axon action potential firing is coincident with highly localized rises in dendritic fluorescence found at the axonal interface (Fig 11Ai, Cii-iii). However, to record a signal above background noise it was necessary to apply a more extended stimulus (30–45 Hz for 10–32 stimuli; Fig. 11Aii, n  = 5).

### Synaptically Localized Activation of K_Ca_2 Channels

**Figure 12:**
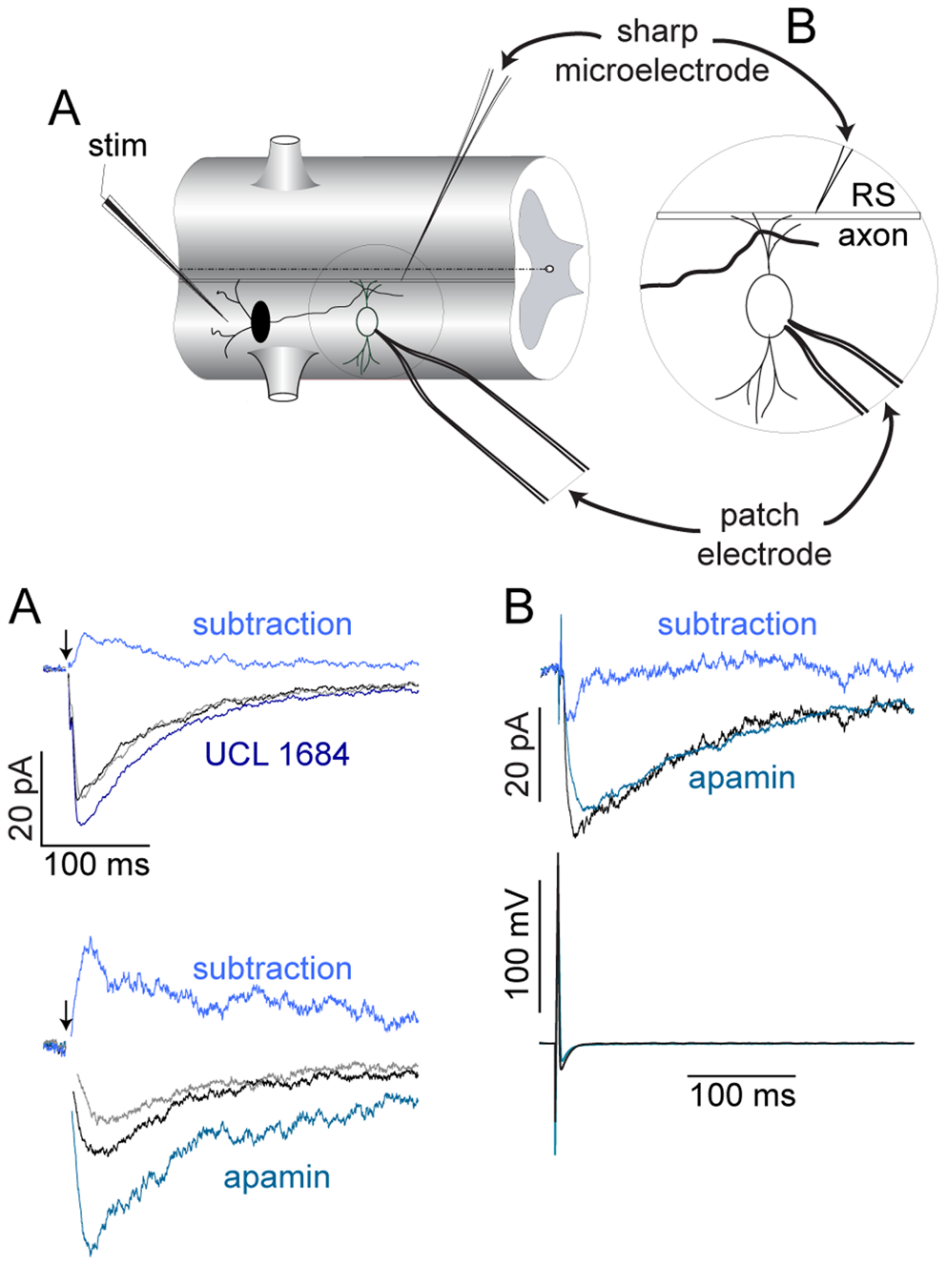
Synapse-dependent K_Ca_2 channel activation. **A–B** Recording schematic depicting how responses were presynaptically evoked and recorded postsynaptically in ventral horn neurons in whole-cell patch clamp configuration using either extracellular EIN (A) or paired intracellular RS axon (B) stimulation. Average NMDA receptor EPSCs were recorded in voltage clamp at −60 mV (A, EIN) and −70 mV (B, RS) after removal of extracellular Mg^2+^ in control (black, 5 µM NBQX, 1 µM glycine and 5 µM strychnine), drug (100 nM UCL 1684, dark blue; 5 µM apamin, teal), and washout (grey) conditions. Corresponding subtractions for EIN-evoked responses reveal the underlying K_Ca_2 current (A, top traces; light blue for UCL and apamin). Paired NMDA receptor EPSCs (B, top trace) from single RS axon action potentials (B, bottom trace) before (black) and after apamin (teal; 5 µM). Subtraction reveals no underlying K_Ca_2 current (top trace, blue). Arrows denote time of single EIN stimulation. Stim = stimulating electrode.

Both EIN and RS axons evoke Ca^2+^ transients in CPG neurons. Thus, we compared input from RS neurons to responses from EINs to determine whether these evoked Ca^2+^ signals lead to K_Ca_2 channel activation. If Ca^2+^ entry via NMDA receptors at glutamatergic synapses is sufficient to activate K_Ca_2 channels, then EPSCs will be augmented by apamin by blocking an evoked outward current caused by synaptically evoked Ca^2+^ entry. Paired cell recordings were made between a presynaptic RS axon using a sharp microelectrode and a postsynaptic neuron recorded in whole-cell voltage clamp. Postsynaptic neurons were held at −60 mV, Mg^2+^ was removed from the superfusate and NBQX (5 µM), and strychnine (5 µM) added to isolate NMDA receptor EPSCs and glycine (1 µM) to ensure NMDA receptor activation. Superfusion of apamin (5 µM) over the preparation had no significant effect on the amplitude of single, monosynaptic RS-evoked NMDA receptor EPSCs (Fig. 12B; P>0.05, n  = 10).

To determine if EIN-evoked NMDA receptor EPSCs activate K_Ca_2 channels. Evoked NMDA receptor EPSCs were achieved as before with an extracellular stimulating electrode in the presence of NBQX (5 µM), glycine (1 µM) and strychnine (5 µM) at a holding potential between −60 and −50 mV. Both apamin and the potent K_Ca_2 channel inhibitor, UCL 1684, led to a significant and reversible increase of EPSC amplitude (Fig. 12A; 52±15%, P<0.01, n  = 8 in 5 µM apamin; 9.1±4.3% from control, P<0.05, n  = 7 in 100 nM UCL 1684). This demonstrates that EIN-evoked NMDA receptor EPSCs leads to Ca^2+^ entry sufficient to activate K_Ca_2 channels necessary for the repolarization phase of the oscillation. Indeed, the components necessary for membrane potential oscillations are synaptically located.

## Discussion

Nonlinear dynamics, such as oscillations, are found in all regions of the nervous system and underlie activities from encephalographic recordings of coordinated brainwave behavior [64], to bursting activity thought to underlie cognition [19]. Within neural networks, Ca^2+^ plays a vital role in the coordination of nonlinear membrane properties generating oscillatory behavior. In spinal premotor and motoneurons, Ca^2+^ entry through the plasma membrane contributes to both depolarization and indirectly to repolarization of neurons through the activation of Ca^2+^-dependent K^+^ currents (i.e. K_Ca_2), tempering excitability and synaptic plasticity [14].

Nonlinear membrane properties in response to NMDA receptor activation and Ca^2+^ entry are central to the generation of membrane potential oscillations in lamprey ventral horn neurons which contribute to the precise rhythmic output of the spinal CPG [63]. Pattern generation requires both intrinsic neuron oscillatory properties [2], and precise synaptic connections for segmental [63], [65]–[67] and intersegmental [68] coordination. If intrinsic oscillatory properties activated by NMDA receptors contribute to locomotion, then synaptically released glutamate is required during rhythmic behavior. Both descending RS command neurons [69]–[71] and local EINs [3] lead to synaptic activation of NMDA receptors. By virtue of Mg^2+^ block [56], NMDA receptors also contribute a voltage-dependent depolarization in addition to Ca^2+^ entry as may VGCCs. However, depending on the location of K_Ca_2 channels with respect to the NMDA receptor [2], VGCC [57] or internal store [72], [73], and the degree of depolarization, Ca^2+^ entry to the cytosol may subsequently activate an outward current. The relationship between this Ca^2+^ entry and secondary effectors will influence oscillatory properties extending from the cell to network level. Thus, it is important to determine the route of Ca^2+^ entry that drives K^+^ current needed to terminate bursting activity [8]. While Ca^2+^ release from internal stores has little or no effect on NMDA-induced fictive locomotion [29], both NMDA receptors and VGCCs are clearly necessary. We have focused on these latter routes of Ca^2+^ entry to determine their contribution to nonlinear properties of neurons during oscillatory activity.

Oscillations evoked in NMDA and TTX demonstrate a remarkably stereotypical value of peak depolarization very close to −40 mV. This is recorded regardless of the oscillation frequency, which is slower than locomotion. However, this slow oscillation frequency is reflective of NMDA-induced fictive swimming which is slower than either brainstem-evoked [1] or AMPA-induced [74] fictive swimming in isolated spinal cords. Additionally, in cells dialyzed with high concentrations of EGTA (5 mM; Fig. 1Ai, B, C), the oscillation is prolonged. When fast Ca^2+^ buffering occurs with BAPTA dialysis (Fig. 1Aii), or when K_Ca_2 channels are blocked directly with UCL 1684 (Fig. 1C), subsequent repolarization may be mediated by voltage-dependent K^+^ conductances [75]. Thus, the variability of the oscillation duration depends on a complex cellular mechanism, which leads it to differ substantially from *in vivo* oscillations in which excitatory and inhibitory synaptic coupling are important.

In non-spiking conditions (i.e. in TTX or subthreshold oscillations during fictive swimming), NMDA receptors and VGCCs may be activated during the depolarization phase of ventral horn neuron oscillations, yet the relative thresholds for VGCC and NMDA receptor activation have not been examined in neurons within the spinal cord. Direct comparisons between the VGCC activation threshold (made by measuring Ba^2+^ currents) and the peak plateau membrane potential reached within the same cell during NMDA-evoked TTX-resistant oscillations revealed a sharp contrast (Fig. 2). Thresholds of voltage-activated Ba^2+^ currents were often more depolarized than the plateau peak (Fig. 2A), whereas NMDA application led to substantial current at less depolarized voltage steps within the membrane potential oscillation range (Fig. 2B, 3B). This demonstrates that VGCCs either remain largely inactive, or just begin to activate as the oscillation reaches its peak. While it is conceivable that even minimal activation of VGCCs would cause K_Ca_2 channel activation due to either close coupling or the low threshold (EC_50_ ∼0.3 µM) for Ca^2+^-dependent K_Ca_2 activation [76], our data suggest that NMDA receptor current is the dominant source of Ca^2+^ whereas VGCCs contribute very little Ca^2+^ within the oscillation range. Ventral horn neurons undergo phase-locked oscillations in Ca^2+^_i_ with the largest amplitudes in distal dendrites [33]. Thus, intracellular Ca^2+^ signals parallel NMDA-induced changes in membrane potential [77], localized to regions of the cell innervated by glutamatergic synapses [35]. Additionally, oscillations occur subthreshold to spiking, while action potentials in the same cells only increased somatic Ca^2+^, leaving dendritic Ca^2+^ oscillation amplitudes unaffected. For these reasons, we hypothesized that NMDA receptors provide the main source of Ca^2+^ underlying the repolarization phase of the oscillation via K_Ca_2 channel activation, localized to dendritic synapses.

During behavior, NMDA receptors are necessarily synaptically activated and require depolarization to allow Ca^2+^ entry, whereas VGCCs can be activated independently of synaptic drive. NMDA receptors and VGCCs will differentially impact the dynamic range of intrinsic membrane properties [12]. Differences in location, voltage-sensitivity, activity-dependency, and kinetic profiles will correspondingly impact dendritic integration and computation [13], [47], [78], [79]. Furthermore, a number of other neuromodulatory systems [54] have been shown to modify these currents. The effectiveness of such metaplastic modulators will depend on their appropriate targeting in spinal neurons. In vertebrates [37] and specifically lamprey ventral horn neurons, VGCC activation following an action potential activates K_Ca_2 channels to cause a late after-hyperpolarization [8], [30], [38], [80]. Yet, the route of Ca^2+^ entry that activates K_Ca_2 currents at voltages subthreshold to action potentials during both locomotion and NMDA-dependent TTX-resistant oscillations has remained elusive. We now show that NMDA causes a profound hyperpolarized shift in the VGCC I-V relationship in ventral horn neurons, leading to activation at lower voltages observed during NMDA-dependent membrane potential oscillations (Figs. 2–5), indicating that primarily NMDA receptor-based Ca^2+^ entry occurs within the membrane potential oscillation range.

We demonstrate that Ca^2+^ entry is located physically close to the K_Ca_2 channels that it activates. Step-evoked NMDA receptor-mediated currents activated a delayed outward current when neurons were dialyzed with EGTA (Fig. 3). Outward currents were prevented in Ba^2+^ (Fig. 1) or BAPTA (Fig. 3), which binds entering Ca^2+^ before binding endogenous buffers [52]. Similarly, BAPTA, but not EGTA, abolishes the NMDA-induced membrane potential repolarization (Fig. 1). These results indicate a close association between K_Ca_2 channels and NMDA receptors and demonstrate the importance of proximity for achieving K_Ca_2 channel activation.

Furthermore, Ca^2+^ imaging experiments corroborates the localized nature of synaptic activation and subsequent Ca^2+^ entry following stimulation of either local EINs (Fig. 10), or RS descending command neurons (Fig. 11). We demonstrate that Ca^2+^ increases are isolated to within short distances (∼10s of microns) around points of entry. However, such synaptic Ca^2+^ entry is capable of activating a substantial apamin- and UCL 1684-sensitive outward current following a single stimulus of presumed EINs (Fig. 12A), but not by RS axon activation (Fig. 12B). Repetitive and single RS axon stimulation during paired cell recording failed to evoke apamin-sensitive EPSPs [53] or currents (Fig. 12B), respectively. Ideally, paired recordings between EINs and ventral horn neurons would clarify whether expressly monosynaptic NMDA receptor EPSCs can activate K_Ca_2 currents, but due to their small size, EINs are difficult to identify and individually stimulate [65].

EINs [3] and RS axons [69]–[71] are both sources of glutamatergic input onto ventral horn neuron dendrites, but serve different functions. RS axons provide descending excitation to spinal CPGs along the length of the spinal cord and thus cannot be phase-locked to multiple segments simultaneously. EINs, however, make local connections within a few consecutive CPG segments and phase-lock to corresponding ventral root discharges [65]. This lack of phase-locked excitation during RS neuron activation perhaps provides one explanation for why RS synaptic activation of Ca^2+^ entry does not directly couple to K_Ca_2 current activation. Such a direct coupling will generate phase-inappropriate hyperpolarization, which would disrupt locomotion. Thus, the close spatial coupling of excitation-evoked Ca^2+^ entry and subsequent outward current activation will correspondingly impact is physiologically important for information processing and coordination of locomotor circuits.

The efficacy of BAPTA at blocking TTX-resistant oscillations and step-evoked late outward currents indicates a close spatial relationship between Ca^2+^ entry and K_Ca_2 channel activation. We also show that EGTA is capable of interfering slightly with repolarization by prolonging the plateau duration during NMDA-dependent TTX-resistant oscillations (Fig. 1B). The latter finding implies that Ca^2+^ diffusing to K_Ca_2 channels beyond the synaptic nanodomain may contribute to oscillatory properties of neurons. Nevertheless, the complete disruption of oscillations in BAPTA (Fig. 1A) demonstrates that the intimate spatial coupling of Ca^2+^ entry with K_Ca_2 activation is necessary for the repetitive oscillations. Thus, oscillations may be achieved through a postsynaptic complex formed by NMDA receptors and K_Ca_2 channels in ventral horn neuron dendrites to drive the cellular oscillatory rhythm. Similar structural arrangements have been reported elsewhere in the CNS [17] and may play an influential role in synaptic modification [14] underlying the short- and long-term modulation of the lamprey CPG.

Our imaging data also demonstrates that the majority of Ca^2+^ entering neurons during NMDA-induced oscillations requires NMDA receptor activation, but not VGCCs. Both Na^+^ and Ca^2+^ permeate the NMDA receptor to cause depolarization. Thus, step-evoked NMDA receptor-mediated currents may not directly reflect an increase in Ca^2+^ entry within the oscillation range. We used Ca^2+^-sensitive dyes, Fluo-5F and OGB1, as reporters of both increases in [Ca^2+^]_i_ and location of Ca^2+^ entry. This allowed a direct comparison of Ca^2+^ entry between VGCCs and NMDA receptors. Over the membrane potential oscillation range (steps to −40mV), very little VGCC-dependent current is recorded and, correspondingly, very little Ca^2+^ dye fluorescence is detected at any somatic or dendritic location (Fig. 4A,C). However, NMDA caused both substantial voltage-dependent current and Ca^2+^ entry over the same voltage range (Fig. 4B–C). This demonstrates that NMDA-induced current correlates well to Ca^2+^ entry within the oscillation range. Inevitable voltage breakthrough resulting from uncorrectable space clamp errors may contribute to this enhancement in NMDA-evoked currents. However, block of VGCCs with ω-CgTxMVIIC, which selectively inhibits N- and P/Q-subtype VGCCs [60] accounting for over 70% of the total VGCC current [57], had little effect on the either membrane potential or Ca^2+^ oscillations (Fig. 8B). Similarly, Cd^2+^ abolishes VGCC current but did not prolong membrane potential oscillations. Thus, because oscillations persist and do not increase in duration, and Ca^2+^-sensitive dye fluorescence does not reduce after pharmacological block of VGCCs, it is unlikely that VGCCs contribute substantially to the repolarization. This suggests that the NMDA receptor, which is maximally activated by the peak of the oscillation, is the primary route of Ca^2+^ entry that subsequently activates K_Ca_2 channels to drive the repolarizing phase of the oscillation.

### Conclusion

Previously, VGCCs have been shown to activate K_Ca_2 in lamprey spinal neurons [8], [30], [38], [80]. Our data demonstrates that there are most likely two sub-populations of apamin-sensitive K_Ca_2 channels [81] which differ both spatially and functionally. One group is activated by ω-CgTxMVIIC-sensitive high voltage activated VGCCs following action potentials, and cause the action potential slow afterhyperpolarization [30], [53] that mediates spike frequency adaptation. The other group contains K_Ca_2 channels located in dendrites, closely apposed to NMDA receptors activated by glutamate release from EINs. These K_Ca_2 channels participate in the repolarization of membrane potential oscillations. The location and route of Ca^2+^ entry is central to the corresponding intrinsic membrane property and subsequent output.

It has proven difficult to sort the physiological roles of various Ca^2+^ ionophores whose activation is voltage-dependent in excitatory, synaptically driven neural behaviors. In the case of spinal motor oscillators, their function requires the activation of NMDA receptors, but Ca^2+^-activated outward currents might arise from a number of distinct systems. We demonstrate that the dominant Ca^2+^ signal for K_Ca_2 channel activation responsible for nonlinear oscillations underlying rhythmogenesis in lamprey ventral horn neurons is entry through the NMDA receptor itself, particularly those receptors synaptically activated by local spinal EINs.
